# The association between habitual emotion regulation, childhood adversity, and cluster C personality disorders symptomatology

**DOI:** 10.3389/fpsyg.2026.1893753

**Published:** 2026-09-03

**Authors:** Ştefania M. Crişan, Andrei C. Miu, Aurora Szentágotai-Tătar

**Affiliations:** 1 Department of Clinical Psychology and Psychotherapy, International Institute for the Advanced Studies of Psychotherapy and Applied Mental Health, Babeş-Bolyai University, Cluj-Napoca, Romania; 2 Cognitive Neuroscience Laboratory, Department of Psychology, Babeş-Bolyai University, Cluj-Napoca, Romania; 3 Center of Excellence for Neuroscience and Mental Health, Babeş-Bolyai University, Cluj-Napoca, Romania

**Keywords:** Avoidant personality disorders, childhood adversity, cluster C personality disorder, Dependent personality disorders, emotion regulation goals, emotion regulation strategies, Obsessive-compulsive personality disorder

## Abstract

**Introduction:**

The mechanisms linking childhood adversity and cluster C personality disorders (PDs) remain unclear. Available research points to emotion regulation as a possible mechanism. Emotion regulation difficulties are documented in the case of cluster C, but studies focus on strategy implementation, while regulation goals received less attention. This study aimed to examine the associations between childhood adversity, emotion regulation goals and strategies, and cluster C PDs. Furthermore, we explore the moderating role of childhood maltreatment in the association between emotion regulation and cluster C.

**Methods:**

This cross-sectional study employs a sample of 141 adults from the Romanian community (aged 18–55 years; *M* = 27.31, SD = 9.13). We employed instruments assessing facets of childhood adversity (CTQ, QUIC, MSPSS, CTSPC, RFQ, and the MacArthur Scale of SES), emotion regulation goals (ERGS) and strategies (E-ERQ), cluster C symptoms (SNAP-2) and associated personality dysfunction (LPFS-BF).

**Results:**

Main analyses suggest significant associations between avoidant PD and total QUIC scores (*r* = 0.206, *p* ≤ 0.05) and total MSPSS scores (*r* = −0.206, *p* ≤ 0.05). Instrumental goals correlated positively with both avoidant PD (*r* = 0.337, *p* ≤ 0.001) and dependent PD (*r* = 0.339, *p* ≤ 0.001). Reappraisal showed negative correlations with avoidant PD (*r* = −0.224, *p* ≤ 0.05) and dependent PD (*r* = −0.219, *p* ≤ 0.05). Avoidant PD was also correlated with suppression (*r* = 0.336, *p* ≤ 0.001) and selective attention (*r* = 0.203, *p* ≤ 0.05). Obsessive-compulsive PD was correlated with situation selection (*r* = 0.200, *p* ≤ 0.05). Importantly, childhood maltreatment moderated the association between hedonic goals and obsessive-compulsive PD. Sensitivity analyses were conducted to account for deviations from assumptions in the main analysis.

**Conclusion:**

Findings highlight the increased use of instrumental goals, suppression, situation selection, selective attention (which could entail disengagement from emotions), and reduced use of reappraisal. Taking the interplay between childhood adversities and emotion regulation difficulties in the case of cluster C into account could expand future literature, which may contribute to the improvement of assessment, prevention, and intervention efforts for cluster C symptoms, especially when exposure to childhood adversities is present.

## 1 Introduction

Cluster C personality disorders (avoidant (AVPD), dependent (DPD), and obsessive-compulsive (OCPD) personality disorders) are characterized by persistent and pervasive cognitive, emotional, interpersonal, and impulse control patterns that diverge from socio-cultural conventions and cause significant impairment in day to day life (American Psychiatric Association, 2013). These personality disorders are chronic and debilitating (Bornstein, 2012; Diedrich and Voderholzer, 2015; Lampe and Malhi, 2018), leading to psychosocial problems (e.g., social withdrawal, submissiveness, prioritizing work over relationships; Bornstein, 2012; Diedrich and Voderholzer, 2015; Lampe, 2016; Kavanagh et al., 2021), and diminished capacity to work (Post et al., 2018; Soeteman et al., 2008). Cluster C personality disorders can be comorbid conditions (e.g., Disney, 2013; Weinbrecht et al., 2016) and present an increased comorbidity with other clinical disorders (such as mood disorders or substance abuse; e.g., Friborg et al., 2014; Goretti et al., 2017; Skodol et al., 2007; Zheng et al., 2019). The literature indicates that, overall, these disorders are frequent, with studies estimating that approximately 5% of the general population presents cluster C symptomatology (Winsper et al., 2020).

Prominent theories (such as the Cognitive Behavior Theory or Schema Theory; Beck et al., 2015; Young et al., 2003) maintain that adverse childhood events (ACEs) play a causal role in the emergence and course of this symptomatology, by maladaptively shaping the way individuals perceive the self, the world, and others (i.e., cognitive schemas). More specifically, through repeated negative childhood experiences characterized by criticism and rejection, individuals learn that they are vulnerable, and the world is harmful (in the case of AVPD), that they are helpless, powerless, and need someone in order to survive (in the case of DPD), or that they are liable and need to control everything and be perfect (in the case of OCPD). These schemas contribute to increased emotional distress and maladaptive coping behaviors, which, in turn, reinforce the maladaptive schemas. These factors interact and reinforce each other, and, in time, they lead to increased vulnerability to AVPD, DPD, and OCPD symptomatology, in the absence of occurrences that contradict the maladaptive schemas (e.g., Beck et al., 2015; Birgenheir and Pepper, 2011; Young et al., 2006).

Empirical studies support these theories, by pointing out an association between a variety of childhood adversities, ranging from childhood maltreatment to household dysfunction, and cluster C PDs (e.g., Johnson et al., 2006; Scheffers et al., 2019; Zhao et al., 2021). More specifically, a recent meta-analysis indicated that childhood adversities are consistently and positively associated both with overall cluster C, and with each specific personality disorder included in this cluster (Crişan et al., 2023). Cross-sectional analyses have pointed out that the prevalence rates of childhood maltreatment among individuals presenting cluster C diagnoses were generally high (Battle et al., 2004). Furthermore, Hengartner et al. (2013) found that adversities such as bullying victimization, emotional abuse, emotional neglect, and conflicts with parents predicted cluster C personality dimensions. Moreover, evidence also stems from longitudinal studies. For example, a prospective longitudinal design that included 593 families suggested that problematic parental behaviors (including low parental care, harsh punishment, or problematic communication with the child) contributed to the risk of developing AVPD, DPD, and OCPD symptomatology in offspring (Johnson et al., 2006). However, the impact of ACEs on cluster C etiology remains unclear, as the data are heterogeneous (e.g., Birgenheir and Pepper, 2011; Johnson et al., 2006; Reising et al., 2019; Scheffers et al., 2019). Moreover, although available theories point to possible mechanisms that explain the impact of childhood adversity on cluster C, empirical research is still necessary.

A prominent line of research suggests that emotion regulation processes are a possible transdiagnostic mechanism mediating the link between childhood adversity and psychopathology (e.g., Dvir et al., 2014; Miu et al., 2022). The literature shows that exposure to childhood adversity influences the development of behavioral and emotional regulatory systems, further leading to an overactive stress-response system and increased sensitivity to threat, that hinder effort to down-regulate emotional experiences (Dvir et al., 2014; Lupien et al., 2009; Miu et al., 2017; Schweizer et al., 2016). In turn, emotional dysregulation contributes to internalizing or externalizing symptoms, and, in the long term, to vulnerability to psychopathology (e.g., Dvir et al., 2014; Gross and Jazaieri, 2014; Hogg et al., 2022; Werner and Gross, 2010).

In the case of cluster C, the evidence supporting the presence of emotion regulation difficulties suggests that AVPD and DPD are associated with nonacceptance of emotional responses, difficulty engaging in goal directed behavior (in the case of DPD), and restricted access to adaptive emotion regulation strategies (Garofalo et al., 2018). In the same study, OCPD showed an association with impulse control problems when faced with emotional distress (Garofalo et al., 2018). Other studies have suggested that AVPD and OCPD are associated with experiential avoidance and overcontrol of emotions, while DPD is negatively associated with suppression (Borges and Naugle, 2017; Wheaton and Pinto, 2017; Weinbrecht et al., 2016). Furthermore, in an experimental study, participants with AVPD presented elevated levels of anxiety while anticipating the use of reappraisal during an emotion regulation task (Denny et al., 2015). Previous investigations that compared borderline PD with cluster C found that the presence of childhood maltreatment and comorbid personality disorders impacts personality functioning and emotion regulation associated with cluster C, even though these disturbances are more severe for borderline PD (Aleknaviciute et al., 2016; Gratz et al., 2013; Snir et al., 2017; Steenkamp et al., 2015; van Zutphen et al., 2018). As such, there is evidence linking childhood adversity and cluster C, and broad emotion regulation difficulties and cluster C symptomatology. However, existing research has focused on these isolated pathways and has not addressed or examined these variables together, in the same model. This gap in the literature limits understanding of the mechanisms through which childhood adversity contributes to the development and maintenance of cluster C pathology.

According to Gross (1998, 2015), emotion regulation implies attempting to influence emotions, their experience and their expression, using several steps: the identification stage (determining whether regulation is necessary); the selection stage (activation of emotion regulation goals and selection of regulation strategy); the stage of strategy implementation; and monitoring of regulation strategies (maintaining, changing, or stopping emotion regulation). This model is not validated in the case of cluster C. Studies so far indicate that cluster C is associated with emotion regulation strategies that lead to negative outcomes in the case of other types of psychopathology (see, for example, Aldao et al., 2010; Prefit et al., 2019), research thus focusing on the implementation stage. How emotion regulation difficulties manifest in the case of cluster C personality disorders, how they impact the course, symptomatology and impairment of this cluster, and how emotion regulation fares as a factor in the association between childhood adversities and these personality problems remains unknown.

A venue that gained attention in the case of other personality disorders (López-Pérez and McCagh, 2020; Millgram et al., 2020), but received little attention in the case of cluster C refers to emotion regulation goals (Mauss and Tamir, 2014). While emotion regulation strategies refer to the ways in which people regulate emotions, emotion regulation goals refer to the reasons why these strategies are employed (Gross, 2015; Mauss and Tamir, 2014). More specifically, emotion regulation goals are defined as representations of a desired emotional state, that motivate the direction of regulatory efforts (Mauss and Tamir, 2014; Millgram et al., 2015). Examining and understanding emotion regulation goals in the case of cluster C is both theoretically and clinically warranted. Cluster C personality disorders are characterized by avoidance (in the case of AVPD), interpersonal dependency (in the case of DPD), and by overcontrol and rigidity (in the case of OCPD) (Bornstein, 2020; Diedrich and Voderholzer, 2015; Disney, 2013; De Reus and Emmelkamp, 2012; Lampe and Malhi, 2018; Weinbrecht et al., 2016). These features could indicate that individuals with cluster C may pursue specific emotion regulation goals (such as minimizing negative emotions in the case of AVPD, impression management and persevering in relationships in the case of DPD, or achieving emotional control to prevent failure in the case of OCPD) that are likely to influence strategy selection and contribute to the maintenance of cognitive schemas and cluster C symptomatology. Clarifying the motivational processes behind the identified emotion regulation difficulties could have important implications for the clinical picture of cluster C and could inform more targeted interventions.

Emotion regulation goals are categorized as hedonic and instrumental motives (Eldesouky and English, 2019a; Mauss and Tamir, 2014; Tamir, 2016). Hedonic motives aim to change an emotional state, either by feeling more positively (pro-hedonic goals), or by feeling more negatively (contra-hedonic goals). On the other hand, instrumental motives target the benefits of emotions, beyond their hedonic experience. Instrumental goals aim at performing and obtaining an outcome from an activity (performance goals), at relationships or social interaction with others (pro-social goals), or to managing one's appearance in front of others (impression management goals; Eldesouky and English, 2019a; Tamir, 2016).

In this study, we aim to investigate the associations between ACEs, habitual emotion regulation goals and strategies, cluster C and associated disturbances in personality functioning. A review conducted by Millgram et al. (2020) shows what emotion regulation goals drive regulatory efforts in the case of psychopathology and proposes how these regulation goals manifest in the case of cluster B personality disorders. This research venue received attention in the case of borderline PD (López-Pérez and McCagh, 2020), but no studies were conducted on emotion regulation goals in the case of cluster C.

Studies indicate that these regulation goals are associated with and predict the use of regulation strategies (Eldesouky and English, 2019b). Concretely, pro-hedonic goals are associated with use of situation selection, situation modification, reappraisal, and distraction, while the use of suppression is associated with impression management goals. Contra-hedonic goals correlated positively with suppression and negatively with reappraisal. Consequently, pro-social goals are associated with the use of distraction and reappraisal, while impression management goals are associated with lower use of reappraisal (Eldesouky and English, 2019b). Studies also suggest that emotion regulation goals and strategies vary as a function of personality (Aldao, 2013; Eldesouky and English, 2019a). It is, however, unclear how emotion regulation goals and strategies, measured at one point in time, are associated when cluster C symptomatology is present. Addressing this gap would extend both the emotion regulation model posited by Gross (1998, 2015) and inform existing theories on cluster C development and maintenance, by testing whether emotion regulation processes serve as a mechanism linking exposure to childhood adversity and cluster C symptomatology. Compared to other personality disorders, cluster C remains relatively understudied, although highly debilitating (e.g., Diedrich and Voderholzer, 2015; Disney, 2013; Lampe and Malhi, 2018). This study aims to provide the first empirical exploration of these associations in a sample presenting cluster C symptoms. We expect to find significant associations between emotion regulation goals and strategies, similar to previous studies.

When it comes to the association between regulation strategies and cluster C, the literature on the implementation stage is more abundant. Studies report that these disorders are associated with suppression (Borges and Naugle, 2017) and that individuals with AVPD symptoms present anticipatory anxiety when it comes to implementing reappraisal (Denny et al., 2015). Considering that the use of reappraisal is negatively associated with psychopathology, while suppression is positively associated with clinical disorders (Aldao et al., 2010; Miu et al., 2022; Prefit et al., 2019) we expected that AVPD, DPD, and OCPD symptomatology be significantly associated with suppression and reappraisal.

Referring to childhood adversity, studies indicate significant associations with cluster C (e.g., Battle et al., 2004; Birgenheir and Pepper, 2011; Johnson et al., 2006), and report that childhood adversity contributes to impairments in functionality in the case of this cluster (Aleknaviciute et al., 2016; Massaal-van der Ree et al., 2022). The literature is, however, scarce on the association of cluster C with a wider array of ACEs (such as poverty or social support; Finkelhor et al., 2015). We propose to investigate whether extended forms of childhood adversity (such as childhood maltreatment, unpredictability during childhood, perceived social support, familial risk, and socio-economic status during childhood) are associated with these personality disorder symptoms. In what concerns the link between childhood adversity and habitual emotion regulation, the literature suggests that hedonic goals are significantly associated with childhood maltreatment, while instrumental goals are not (Ion et al., 2023). Important reports suggest that childhood maltreatment has also been positively associated with reappraisal, and negatively associated with rumination and suppression (Ion et al., 2023; Miu et al., 2022). We expected that the results of previous studies would be replicated in our study.

Little is known on how childhood maltreatment and habitual emotion regulation contribute to cluster C symptomatology. We propose to explore their shared contribution to cluster C by investigating whether childhood maltreatment moderates the associations between habitual emotion regulation goals (hedonic and instrumental) and strategies (reappraisal, suppression, distraction, selective attention, and situation selection) and cluster C symptoms.

To summarize, this study investigated the following hypotheses:

1. Facets of childhood adversity (i.e., childhood maltreatment, unpredictability, socio-economic status, familial risk and perceived social support during childhood) are significantly associated with AVPD, DPD, OCPD symptoms, and personality dysfunction.
2. AVPD, DPD, and OCPD symptoms are significantly associated with reappraisal and suppression.
3. Childhood maltreatment is significantly associated with hedonic emotion regulation goals.
4. Childhood maltreatment is significantly associated with reappraisal and suppression.
5. Emotion regulation goals (i.e., hedonic and instrumental goals) and strategies (i.e., reappraisal, suppression, distraction, selective attention, and situation selection) are significantly associated.
6. Childhood maltreatment moderates the associations between habitual emotion regulation goals (hedonic and instrumental) and strategies (reappraisal, suppression, distraction, selective attention, and situation selection) and AVPD, DPD, and OCPD symptoms.

## 2 Methods

### 2.1 Study design

This research was pre-registered in OSF (https://osf.io/e3b6r). This study includes a cross-sectional, correlational design.

### 2.2 Participants

Three hundred and twenty-six Romanian participants enrolled in this study. Participants were included if they had a minimum age of 18 years and if they presented symptoms of at least one cluster C personality disorder (measured using the Schedule for Nonadaptive and Adaptive Personality - 2nd Edition [SNAP-2]; Clark et al., 2014). Out of the 326 enrolled participants, 185 participants (56.7%) were excluded for the reasons described in Table 1. To examine potential selection bias, excluded and included participants were compared on age and sex variables. Excluded participants differed from included participants in terms of age (*t* (324) = −2.48, *p* = 0.014, Mean difference = −2.75, 95% CI = - 4.937, - 0.569), but did not differ in terms of sex ( χ^2^ (1) = 2.25, *p* = 0.133). The final sample (*N* = 141) consisted of 125 females (88.7%) and 16 males (11.3%), with ages ranging from 18 to 55 years old (*M* = 27.31, SD = 9.13) and was significantly younger than excluded participants.

**Table 1:** Descriptives of the initial sample (*N* = 326).

Sample information	*N*	% of initial sample	*M*_*age*_ (SD)	*N*_*Females*_ (%)
Initially enrolled	326	100		
Excluded participants	185	56.7	30.06 (10.49)	153 (46.93)
Duplicate registration	1	0.3		
Age < 18	1	0.3		
Standalone cluster A symptoms	20	6.1		
Standalone cluster B symptoms	9	2.8		
Mix of cluster A and B symptoms	5	1.5		
No personality disorder symptoms	149	45.7		
Included participants	141	43.3	27.31 (9.13)	125 (38.34)

M, mean; SD, standard deviation.

### 2.3 Measures

#### 2.3.1 Schedule for nonadaptive and adaptive personality - 2nd edition (SNAP-2)

The SNAP-2 (Clark et al., 2014) is a 390-item self-report instrument assessing personality traits and symptoms from the dimensional and categorical perspectives of personality disorders. The full-length adult version was employed in this study. The items are endorsed as True (1) or False (2). Example items for cluster C personality disorder symptoms subscales include: “I hate it when the topic of conversation turns to me”, “I let other people make important decisions for me”, and “I believe in playing strictly by the rules”. The instrument yields scores on 17 personality trait subscales (e.g., Detachment, Negative temperament), 7 subscales for validity indexes (e.g., Variable response inconsistency, Desirable response inconsistency), and personality disorder symptom scales based on the Diagnostic and Statistical Manual of Mental Disorders - IVth Edition [DSM-IV] (American Psychiatric Association, 1994). The presence of cluster C personality disorders was determined using a combination of elevated norm-referenced *T*-scores (≥60) on relevant personality traits (e.g., detachment and negative temperaments for AVPD; dependency for DPD; and workaholism, propriety, and low disinhibition for OCPD), alongside the number of endorsed diagnostic criteria for each personality disorder included in cluster C. Following participant inclusion, raw scores on the cluster C personality disorders subscales were used as continuous variables in the statistical analyses. In the current sample, internal consistency was acceptable for cluster C personality disorder subscales, with Cronbach α ranging from 0.63 (OCPD subscale) to 0.87 (for the AVPD and DPD subscales).

#### 2.3.2 Level of personality functioning scale - Brief form (LPFS-BF)

The LPFS-BF (Bach and Hutsebaut, 2018) is a 12 item instrument that assesses personality impairment, according to the DSM-5 Alternative Model for Personality Disorders (American Psychiatric Association, 2013). The scale measures overall personality dysfunction, as well as two other domains of personality functioning: individual (identity and self-direction) dysfunction, and interpersonal (empathy and intimacy) dysfunction. Items are rated on a Likert scale ranging from 0 (Very false or often false) to 3 (Very true or often true). Items include statements such as “I often do not know who I really am”, “I often make unrealistic demands on myself”, and “My relationships and friendships never last long”. Scores are obtained by summing the items within each subscale and for the total scale (with possible overall scores ranging from 0 to 36). In the present study, the scores were used as continuous variables and no clinical cut-off was applied. This scale demonstrated good internal consistency, with Cronbach α of 0.80 for the Individual dysfunction subscale, 0.63 for the Interpersonal dysfunction subscale, and 0.80 for the Overall personality dysfunction.

#### 2.3.3 Childhood trauma questionnaire - Short form (CTQ-SF)

This instrument assesses the severity of childhood maltreatment, including the following subscales: emotional abuse, physical abuse, sexual abuse, emotional neglect, physical neglect, and overall maltreatment (Bernstein et al., 2003). The instrument employs 28 items rated on a Likert scale ranging from 1 (Never true) to 5 (Often very true). Examples items include “I felt that someone in my family hated me”, “People in my family hit me so hard that it left me with bruises or marks”, and “I didn't have enough to eat”. Scores are obtained by summing the items within each subscale and for the total score, with some items being reverse coded. In the present study, this instrument was used to assess retrospective exposure to childhood maltreatment, and scores were used as continuous variables. No clinical cut-off was applied. For this instrument, Cronbach α ranged from 0.60 (Physical neglect subscale) to 0.92 (Overall childhood maltreatment).

#### 2.3.4 Questionnaire of unpredictability in childhood (QUIC)

QUIC (Glynn et al., 2019) is an instrument that measures the degree of unpredictability experienced during childhood. It assesses several domains, such as parental monitoring, parental environment, parental predictability, physical environment, and safety and security. The items are rated in a Yes (1) or No (0) format. Scores are obtained by summing up the items within each subscale mentioned above, and for the total score. Examples of items include statements such as “Prior to the age of 12, my parents were often late to pick me up (e.g., from school, aftercare or sports)”, “Prior to the age of 18, at least one of my parents had punishments that were unpredictable”, and “I changed schools frequently”. Some items are reverse coded. The scores were used as continuous variables and no clinical cut-off was applied. We obtained Cronbach α ranging from 0.56 (Safety and security subscale) to 0.85 (Overall unpredictability during childhood) for this instrument.

#### 2.3.5 Multidimensional scale of perceived social support (MSPSS)

This scale is a 12-item instrument measuring perceived social support from three sources: family, friends, and significant others (Zimet et al., 1988). In the present study, we adapted the items to refer to participants' experiences prior to the age of 18. Items are rated on a Likert scale ranging from 1 (Strongly disagree) to 7 (Strongly agree). Examples of items include “I could talk about my problems with my family”, “My friends really tried to help me”, and “I had a special person who was a real source of comfort for me”. Scores are obtained by averaging the items on each subscale and the total score. No items were reverse coded, and no clinical cut-off was applied. Scores were employed as continuous variables indicating perceived social support during childhood. Cronbach α values obtained for this instrument ranged from 0.92 (Perceived support from friends subscale) to 0.96 (Perceived support from significant others subscale).

#### 2.3.6 Conflict-tactics scale - Parent–Child version (CTSPC)

The CTSPC (Straus et al., 1998) is a 29 item self-report instrument that measures parental behaviors and maltreatment frequency experienced during childhood. It includes the following subscales: non-violent discipline, psychological aggression, physical assault, neglect, and sexual abuse. In the present study, the items were rated on a Likert scale ranging from 0 (Never) to 4 (Very often). Items were summed for each subscale, with no items being reverse coded. Examples of items include: “Explained why something was wrong”, “Shouted, yelled, or screamed at”, and “Spanked on the bottom with bare hand”. Scores were employed as continuous variables, with no cut-off being applied. This instrument presented Cronbach α indexes that ranged from 0.41 (Non-violent discipline subscale) to 0.90 (Physical assault subscale).

#### 2.3.7 Risky families questionnaire (RFQ)

RFQ (Taylor et al., 2004) is a self-report instrument that measures risky family characteristics and environment during childhood. It consists of 13 questions rated on a Likert scale ranging from 1 (Not at all) to 5 (Very often). Scores are obtained by summing the items, with some items being reverse coded. Examples of questions employed in this instrument include “How often did a parent or other adult in the household make you feel that you were loved, supported, and cared for?”, “How often did a parent or other adult in the household push, grab, shove, or slap you?”, and “How often would you say there was quarreling, arguing, or shouting between your parents?”. In the present study, scores were employed as continuous variables and no cut-off was applied. We obtained a Cronbach α of 0.87 for this instrument.

#### 2.3.8 MacArthur scale of socio-economic status

This instrument measures subjective social status, both during childhood (up to the age of 12), and during adulthood, at the moment of enrolment (Adler and Stewart, 2007). It consists of 8 items formulated as questions. For the assessment of childhood social status, participants provided information relating to parents' level of education and occupation, as well as ratings of perceived socio-economic status during childhood on a Likert scale ranging from 1 (low socio-economic status) to 10 (high socio-economic status). In the present study, the subjective ratings of socio-economic status during childhood were employed as a continuous variable. No clinical cut-off was applied.

#### 2.3.9 Emotion regulation goals scale (ERGS)

This instrument is a self-report instrument measuring emotion regulation goals (Eldesouky and English, 2019a). It assesses hedonic goals (composed of the pro-hedonic and contra-hedonic goals subscales), and instrumental goals (composed of the performance, pro-social, and impression management goals subscales). The scale consists of 18 questions rated on a Likert scale ranging from 1 (Never) to 7 (Always). Examples of items from this scale include: “When you are regulating your emotions, how often do you do so because you want… ‘To feel less negative emotion?', ‘To keep feeling positive emotion?', ‘To make someone else feel good?”'. Items for each subscale were averaged, with no reverse coding. After averaging the subscale scores, we computed the scores for overall (hedonic and instrumental) emotion regulation goals. The resulting scores were employed as continuous variables. No clinical cut-off was applied. Cronbach α for this scale ranged from 0.28 (Hedonic goals subscale) to 0.88 (Impression management subscale).

#### 2.3.10 Extended emotion regulation questionnaire (E-ERQ)

E-ERQ (Guassi Moreira et al., 2021) is a self-report instrument that measures the use of emotion regulation strategies, such as reappraisal, distraction, suppression, selective attention, and situation selection. This instrument employs 22 items rated on a Likert scale ranging from 1 (Strongly disagree) to 7 (Strongly agree). Items include statements like: “When I want to feel a more positive emotion (such as joy), I change what I'm thinking about”, “I keep my emotions to myself”, and “If I do not like how I am feeling, I try to change my surroundings”. Items were averaged for each subscale, with no reverse coding. Scores were used as continuous variables and no cut-off was applied. Cronbach α ranged from 0.73 (Situation selection subscale) to 0.82 (Reappraisal subscale).

### 2.4 Procedure

Participants were recruited through advertisements posted on social media apps (i.e., Facebook and Instagram) and enrolled in the study voluntarily. Informed consent was provided at registration. At enrolment, participants provided demographic information, and completed all the instruments, in the following order: SNAP-2, LPFS-BF, CTQ-SF, MacArthur Scale of Socio-Economic Status, QUIC, MSPSS, CTSPC, RFQ, ERGS, E-ERQ. Prior to data collection, license authorization for SNAP-2 was obtained from the authors (Clark et al., 2014), providing the official scoring formulas to compute subscale scores and individual personality profiles. These profiles were generated for all enrolled participants, to establish eligibility. Following enrolment, individual personality profiles were calculated for all participants, using the official SNAP-2 scoring formulas provided by the authors (Clark et al., 2014). Only participants who met criteria for cluster C personality disorder symptoms (as detailed in the Measures section) were included in this study.

### 2.5 Data analysis

The analysis was conducted using SPSS Statistics, v.30 (IBM Corp, 2024). The association between variables was analyzed using Pearson's correlation (Cohen, 1988), using the guidelines recommended by Gignac and Szodorai (2016) for interpretation: 0.10 suggesting small associations, 0.20 indicating moderate associations, and 0.30 suggesting large associations. The underlying assumptions for correlation were verified. Normality was examined using the Shapiro-Wilk test (Supplementary Table S1), histograms (Supplementary Figure S1), and normal Q-Q plots (Supplementary Figure S2). These results are presented in the Supplementary material 1. The presence of outliers was investigated via boxplots (Supplementary Figure S3 in Supplementary material 2). Linearity and homogeneity of variances were inspected via scatterplots (Supplementary material 3 presents results for AVPD, DPD, and OCPD in Supplementary Figures S4–S6; Supplementary material 4 presents results for the personality dysfunction variables in Supplementary Figures S7–S9; Supplementary material 5 presents results for childhood adversity and emotion regulation, in Supplementary Figures S10–S26). Because several variables (AVPD, DPD, OCPD, Individual and Interpersonal personality dysfunction, Socio-economic status during childhood, Childhood maltreatment, Childhood unpredictability, Childhood perceived social support, Psychological aggression, Physical assault, Neglect, Sexual abuse, Childhood family risk, Instrumental goals, Reappraisal, Distraction, and Situation selection) showed deviations from normality and linearity, Spearman rank-order correlations were also computed as sensitivity analyses (Supplementary Table S2 in Supplementary material 6). Given that there was a significant age difference between included and excluded participants, and that the sample was composed predominantly of females, partial correlation controlling for age and sex were also conducted (Table S3 in Supplementary material 6).

Moderation analyses were conducted using emotion regulation goals (hedonic and instrumental goals) and strategies (reappraisal, suppression, distraction, selective attention, situation selection) as independent variables, employing AVPD, DPD, and OCPD symptoms as dependent variables, while childhood maltreatment (coded as a continuous variable) was used as a moderator. This analysis was conducted using PROCESS Macro for SPSS, v.5.0 (Hayes, 2022). The assumptions of normality, linearity, and homoscedasticity of residuals were examined. Residuals were saved from each model and inspected using histograms, normal Q–Q plots, and scatterplots of residuals against predicted values (Figures S27–S35 in Supplementary material 6). Visual inspection indicated that the residuals were not distributed normally across variables and that the assumptions of linearity and homoscedasticity were not reasonably met for all residuals. Multicollinearity was assessed via variance inflation factors (VIFs) and tolerance statistics, using moderated regression models (Tables S4–S6 in Supplementary material 6). Given that all variables presented moderate to high VIFs levels (>5; range: 7.40–40.56), and that tolerance levels were lower (< 0.20; range: 0.025–0.135) than conventional thresholds, the data indicated potential multicollinearity issues. To account for these deviations, moderation analyses were conducted using Hayes' PROCESS macro (Model 1) with mean-centered predictors, using age and sex as covariates, heteroscedasticity-consistent standard errors (HC3; Davidson and MacKinnon, 1993), and bootstrapped confidence intervals (Tables S7–S9 in Supplementary material 6).

Given that the sample was composed mostly of females (*N* = 125), we also conducted these analyses excluding the male participants (*N* = 16). As such, partial correlations controlling for age in the case of this sample are presented in Table S10 in Supplementary material 6, while moderation analyses using age as a covariate, heteroscedasticity-consistent standard errors (HC3; Davidson and MacKinnon, 1993), and bootstrapped confidence intervals are presented in Tables S11–S13 in Supplementary material 6. The analyses on male participants were not conducted, as the sample was too small (*N* = 16).

## 3 Results

Descriptives of the sample are included in Table 2. Correlation results are presented in Table 3. The following section presents the outcome of this analysis. Sensitivity analyses were conducted, using Spearman rank-order correlations and partial correlations, with age and sex as covariates (for the correlation analysis), while moderation analyses were conducted with mean-centered predictors, age and sex as covariates, heteroscedasticity-consistent standard errors (HC3; Davidson and MacKinnon, 1993), and bootstrapped confidence intervals. These sensitivity analyses are presented in the Supplementary material and are summarized below.

**Table 2:** Descriptives of included participants (*N* = 141).

Variable	*M*	SD
Age (Range: 18–55)	27.31	9.13
SNAP - Avoidant PD	13.90	4.45
SNAP - Dependent PD	8.60	5.12
SNAP - Obsessive - compulsive PD	16.71	3.60
LPFS - Individual personality dysfunction	11.39	4.28
LPFS - Interpersonal personality dysfunction	7.88	3.45
LPFS - Overall personality dysfunction	19.27	6.66
Socio-economic status during childhood	6.06	1.95
CTQ - Childhood maltreatment	52.86	18.29
QUIC - Childhood unpredictability	11.15	6.28
MSPSS - Childhood perceived social support	4.36	1.59
CTSPC - Non-violent discipline	8.04	3.26
CTSPC - Psychological aggression	10.53	5.70
CTSPC - Physical assault	13.02	10.75
CTSPC - Neglect	5.20	3.74
CTSPC - Sexual abuse	0.62	1.02
RFQ - Childhood family risk	32.20	10.99
ERGS - Hedonic goals	3.88	0.77
ERGS - Instrumental goals	5.12	0.97
E-ERQ - Reappraisal	4.80	1.24
E-ERQ - Suppression	4.41	1.31
E-ERQ - Distraction	4.74	1.26
E-ERQ - Selective attention	3.72	1.35
E-ERQ - Situation selection	4.79	1.40

M, mean; SD, standard deviation; SNAP, Schedule for nonadaptive and adaptive personality - 2nd edition; LPFS, Level of personality functioning scale - Brief form; CTQ, Childhood trauma questionnaire - Short form; QUIC, Questionnaire of unpredictability in childhood; MSPSS, Multidimensional scale of perceived social support; CTSPC, Conflict-tactics scale - parent-child version; RFQ, Risky families questionnaire; ERGS, Emotion regulation goals scale; E-ERQ, Extended emotion regulation questionnaire.

**Table 3:** Correlations between variables (*N* = 141).

Variable	AVPD	DPD	OCPD	LPFS individ	LPFS Interpers	LPFS total	SES child	CTQ total	QUIC total	MSPSS total	CTSPC NVD	CTSPC psych aggress	CTSPC physicalassault	CTSPC neglect	CTSPC sexual abuse	RFQ total	ERGS HG	ERGS IG	E-ERQ R	E-ERQ S	E-ERQ D	E-ERQ SA	E-ERQ SS
AVPD	1																						
DPD	0.511^**^	1																					
OCPD	−0.293^**^	−0.281^**^	1																				
LPFS - Individ	0.515^**^	0.552^**^	−0.053	1																			
LPFS - Interpers	0.408^**^	0.311^**^	0.065	0.479^**^	1																		
LPFS - Total	0.542^**^	0.516^**^	0.000	0.891^**^	0.826^**^	1																	
SES child	0.109	−0.020	0.110	0.085	−0.017	0.046	1																
CTQ - Total	0.099	0.074	−0.052	0.153	0.310^**^	0.259^*^	0.010	1															
QUIC - Total	0.206^*^	0.151	0.004	0.238^*^	0.231^*^	0.272^**^	0.064	0.716^**^	1														
MSPSS - Total	−0.206^*^	−0.120	0.086	−0.241^*^	−0.371^**^	−0.347^**^	−0.015	−0.706^**^	−0.578^**^	1													
CTSPC - NVD	−0.087	−0.061	0.144	−0.124	0.083	−0.037	0.080	0.143	0.009	−0.049	1												
CTSPC - Psych aggress	0.103	−0.031	0.067	0.142	0.211^*^	0.201^*^	−0.026	0.772^**^	0.597^**^	−0.613^**^	0.266^**^	1											
CTSPC - Physical assault	−0.006	−0.037	0.083	0.053	0.268^**^	0.173^*^	0.001	0.688^**^	0.413^**^	−0.481^**^	0.457^**^	0.728^**^	1										
CTSPC - Neglect	0.105	0.142	−0.076	0.141	0.178^*^	0.183^*^	0.023	0.684^**^	0.641^**^	−0.597^**^	0.054	0.560^**^	0.450^**^	1									
CTSPC - Sexual abuse	0.111	0.038	−0.031	0.105	0.129	0.135	0.144	0.404^**^	0.303^**^	−0.191^*^	0.065	0.271^**^	0.258^*^	0.253^*^	1								
RFQ - Total	0.135	0.149	−0.067	0.188^*^	0.202^*^	0.225^*^	0.077	0.808^**^	0.761^**^	−0.660^**^	0.045	0.771^**^	0.559^**^	0.700^**^	0.250^*^	1							
ERGS - HG	0.024	0.063	0.035	0.198^*^	0.191^*^	0.226^*^	−0.051	0.015	0.006	0.022	0.207^*^	−0.044	0.101	0.032	0.094	−0.099	1						
ERGS - IG	0.337^**^	0.339^**^	−0.087	0.262^*^	0.081	0.210^*^	0.030	0.071	0.063	0.013	0.109	0.072	0.081	0.084	0.119	0.075	0.197^*^	1					
E-ERQ - R	−0.224^*^	−0.219^*^	0.110	−0.275^**^	−0.080	−0.218^*^	−0.098	−0.194^*^	−0.134	0.206^*^	0.052	−0.237^*^	−0.123	−0.126	0.068	−0.221^*^	0.131	0.114	1				
E-ERQ - S	0.336^**^	0.142	0.010	0.247^*^	0.336^**^	0.333^**^	−0.047	0.133	0.173^*^	−0.220^*^	0.097	0.129	0.127	0.096	0.063	0.099	0.169^*^	0.271^**^	0.147	1			
E-ERQ - D	0.143	0.111	−0.008	0.106	0.175^*^	0.159	−0.072	0.014	0.077	−0.011	0.060	0.032	−0.020	−0.048	0.106	−0.011	0.130	0.323^**^	0.522^**^	0.378^**^	1		
E-ERQ - SA	0.203^*^	0.161	0.058	0.200^*^	0.313^**^	0.290^**^	0.001	−0.051	−0.002	0.011	0.188^*^	−0.040	−0.008	−0.046	0.098	−0.095	0.210^*^	0.266^**^	0.275^**^	0.387^**^	0.475^**^	1	
E-ERQ - SS	−0.139	−0.094	0.200^*^	−0.020	0.054	0.015	−0.024	−0.097	−0.019	0.084	0.121	−0.052	−0.062	−0.074	−0.015	−0.123	0.287^**^	0.233^*^	0.538^**^	0.111	0.461^**^	0.314^**^	1

^*^p ≤ 0.05; ^**^p ≤ 0.001. AVPD, Avoidant personality disorder; DPD, dependent personality disorder; OCPD, obsessive-compulsive personality disorder; LPFS - Individ, individual personality dysfunction; LPFS - Interpers, interpersonal personality dysfunction; LPFS - Total, overall personality dysfunction; SES child, socio-economic status during childhood; CTQ - Total, childhood maltreatment; QUIC - Total, childhood unpredictability; MSPSS - Total, childhood perceived social support; CTSPC NVD, non-violent discipline; CTSPC - Psych aggress, psychological aggression; RFQ - Total, childhood family risk; ERGS - HG, hedonic goals; ERGS - IG, instrumental goals; E-ERQ - R, reappraisal; E-ERQ - S, suppression; E-ERQ - D, distraction; E-ERQ - SA, Selective attention; E-ERQ - SS, situation selection.

### 3.1 AVPD

#### 3.1.1 Main analysis

We found that AVPD correlated positively with DPD (*r* = 0.51, *p* ≤ 0.001), and negatively with OCPD (*r* = −0.29, *p* ≤ 0.001). Significant associations were also found for this disorder and the subscales of LPFS: individual (*r* = 0.51, *p* ≤ 0.001), interpersonal (*r* = 0.40, *p* ≤ 0.001), and overall personality dysfunction (*r* = 0.54, *p* ≤ 0.001). AVPD was significantly associated with childhood unpredictability (measured with QUIC), *r* = 0.20, *p* = 0.014, and with perceived childhood social support (measured using the MSPSS), *r* = −0.20, *p* = 0.014. No other significant associations were found between AVPD and childhood adversity. In regards to emotion regulation, results indicated significant associations between AVPD and instrumental goals (*r* = 0.33, *p* ≤ 0.001), reappraisal (*r* = −0.22, *p* = 0.008), suppression (*r* = 0.33, *p* ≤ 0.001), and selective attention (*r* = 0.20, *p* = 0.016).

#### 3.1.2 Sensitivity analyses

Spearman's correlation indicated that AVPD correlated positively with DPD (*r*_*s*_= 0.45, *p* ≤ 0.001), and negatively with OCPD (*r*_*s*_= −0.28, *p* ≤ 0.001). Significant associations were found between AVPD and LPFS subscales: individual (*r*_*s*_= 0.44, *p* ≤ 0.001), interpersonal (*r*_*s*_= 0.39, *p* ≤ 0.001), and overall personality dysfunction (*r*_*s*_= 0.50, *p* ≤ 0.001). In terms of habitual emotion regulation, this type of pathology correlated with instrumental goals (*r*_*s*_= 0.27, *p* = 0.001), reappraisal (*r*_*s*_= −0.25, *p* = 0.002), suppression (*r*_*s*_= 0.29, *p* ≤ 0.001), and selective attention (*r*_*s*_= 0.20, *p* = 0.014). No other significant correlations were found in the case of this sensitivity analysis.

### 3.2 DPD

#### 3.2.1 Main analysis

DPD symptomatology was negatively associated with OCPD (*r* = −0.28, *p* ≤ 0.001), and positively associated with LPFS subscales: individual (*r* = 0.55, *p* ≤ 0.001), interpersonal (*r* = 0.31, *p* ≤ 0.001), and overall personality dysfunction (*r* = 0.51, *p* ≤ 0.001). No significant associations were identified between this symptomatology and childhood adversity. Referring to emotion regulation, results suggest that DPD was positively associated with instrumental goals (*r* = 0.33, *p* ≤ 0.001), and negatively associated with reappraisal (*r* = −0.21, *p* = 0.009).

#### 3.2.2 Sensitivity analyses

Spearman's correlation indicated that DPD correlated negatively with OCPD (*r*_*s*_= −0.26, *p* = 0.002). Significant associations were found between DPD and LPFS subscales: individual (*r*_*s*_= 0.53, *p* ≤ 0.001), interpersonal (*r*_*s*_= 0.30, *p* ≤ 0.001), and overall personality dysfunction (*r*_*s*_= 0.50, *p* ≤ 0.001). Instrumental goals positively correlated with DPD (*r*_*s*_= 0.31, *p* ≤ 0.001), while reappraisal negatively correlated with this personality pathology (*r*_*s*_= −0.21, *p* = 0.010). No other significant correlations were found in the case of this sensitivity analysis.

### 3.3 OCPD

#### 3.3.1 Main analysis

Results indicated that OCPD did not correlate significantly with any aspect of personality dysfunction or childhood adversity. In terms of emotion regulation, a significant association was found solely between OCPD and situation selection (*r* = 0.20, *p* = 0.017).

#### 3.3.2 Sensitivity analyses

Spearman's correlation indicated that OCPD positively correlated with situation selection (*r*_*s*_= 0.23, *p* = 0.005). No other significant correlations were found in the case of this sensitivity analysis.

### 3.4 Childhood adversity

#### 3.4.1 Main analysis

Childhood adversity was significantly related to personality dysfunction, as measured by the LPFS (see Table 3). We also found significant associations between childhood maltreatment (measured using the CTQ) and habitual use of reappraisal (*r* = −0.19, *p* = 0.021), between childhood unpredictability (measured with QUIC) and habitual use of suppression (*r* = 0.17, *p* = 0.040), and between perceived childhood social support (measured with MSPSS), reappraisal (*r* = 0.20, *p* = 0.014) and suppression (*r* = −0.22, *p* = 0.009). Non-violent discipline (measured with CTSPC) was associated with hedonic goals, *r* = 0.20, *p* = 0.014, and selective attention, *r* = 0.18, *p* = 0.026, while psychological aggression (measured by the CTSPC, *r* = −0.23, *p* = 0.005) and childhood family risk (measured using RFQ, *r* = −0.22, *p* = 0.008) were associated with habitual use of reappraisal.

#### 3.4.2 Sensitivity analyses

Spearman's correlation also indicated that several measures of childhood adversity correlated significantly with the LPFS subscales (see Supplementary Table S2). Non-violent discipline (measured with CTSPC) was significantly correlated with hedonic goals (*r*_*s*_= 0.17, *p* = 0.039). Reappraisal correlated with childhood maltreatment (measured with CTQ, *r*_*s*_= −0.20, *p* = 0.017), perceived social support during childhood (measured with MSPSS, *r*_*s*_= 0.18, *p* = 0.031), psychological aggression (measured with CTSPC, *r*_*s*_= −0.21, *p* = 0.009), and childhood family risk (measured with RFQ, *r*_*s*_= −0.21, *p* = 0.011). Suppression was associated with perceived social support during childhood (measured with MSPSS, *r*_*s*_= −0.19, *p* = 0.021).

### 3.5 Habitual emotion regulation

#### 3.5.1 Main analysis

Hedonic goals were associated with individual dysfunction (*r* = 0.19, *p* = 0.018), with interpersonal dysfunction (*r* = 0.19, *p* = 0.023), and with overall personality impairment (*r* = 0.22, *p* = 0.007), measured by the LPFS. There were significant associations also identified between hedonic goals and the use of suppression (*r* = 0.16, *p* = 0.045), selective attention (*r* = 0.21, *p* = 0.013), and situation selection (*r* = 0.28, *p* ≤ 0.001). Instrumental goals were significantly associated with individual, *r* = 0.26, *p* = 0.002, and overall personality dysfunction, *r* = 0.21, *p* = 0.012, measured by the LPFS. There were also positive associations with suppression (*r* = 0.27, *p* ≤ 0.001), distraction (*r* = 0.32, *p* ≤ 0.001), selective attention, (*r* = 0.26, *p* ≤ 0.001), and situation selection, (*r* = 0.23, *p* = 0.006).

#### 3.5.2 Sensitivity analyses

Spearman's correlation indicated that individual personality dysfunction was significantly correlated with hedonic goals (*r*_*s*_= 0.22, *p* = 0.007), instrumental goals (*r*_*s*_= 0.24, *p* = 0.003), reappraisal (*r*_*s*_= −0.26, *p* = 0.001), suppression (*r*_*s*_= 0.25, *p* = 0.002), and selective attention (*r*_*s*_= 0.18, *p* = 0.028). Interpersonal personality dysfunction was associated with suppression (*r*_*s*_= 0.30, *p* ≤ 0.001), distraction (*r*_*s*_= 0.19, *p* = 0.020), and selective attention (*r*_*s*_= 0.35, *p* ≤ 0.001). Overall personality dysfunction was associated with hedonic goals (*r*_*s*_= 0.23, *p* = 0.006), instrumental goals (*r*_*s*_= 0.19, *p* = 0.018), reappraisal (*r*_*s*_= −0.21, *p* = 0.010), suppression (*r*_*s*_= 0.31, *p* ≤ 0.001), distraction (*r*_*s*_= 0.16, *p* = 0.047), and selective attention (*r*_*s*_= 0.29, *p* ≤ 0.001). We also found positive associations between hedonic goals, selective attention (*r*_*s*_= 0.19, *p* = 0.024), and situation selection (*r*_*s*_= 0.23, *p* = 0.006). Instrumental goals were associated with suppression (*r*_*s*_= 0.26, *p* = 0.001), distraction (*r*_*s*_= 0.33, *p* ≤ 0.001), selective attention (*r*_*s*_= 0.22, *p* = 0.007), and situation selection (*r*_*s*_= 0.23, *p* = 0.006).

### 3.6 Partial correlations

Partial correlation analysis indicated that participant age correlated with individual personality dysfunction (*r* (139) = −0.24, *p* = 0.003), childhood unpredictability (measured with QUIC, *r* (139) = −0.17, *p* = 0.038), and non-violent discipline (measured with CTSPC, *r* (139) = 0.22, *p* = 0.008). Participant sex correlated solely with physical assault (measured with CTSPC, *r* (139) = 0.17, *p* = 0.041).

#### 3.6.1 AVPD

AVPD correlated positively with DPD (*r* (137) = 0.53, *p* ≤ 0.001), and negatively with OCPD (*r* (137) = −0.29, *p* ≤ 0.001). Significant associations were found between AVPD and LPFS subscales: individual (*r* (137) = 0.49, *p* ≤ 0.001), interpersonal (*r* (137) = 0.44, *p* ≤ 0.001), and overall personality dysfunction (*r* (137) = 0.54, *p* ≤ 0.001). This type of pathology also correlated with childhood unpredictability (measured with QUIC, *r* (137) = 0.19, *p* = 0.023), and perceived social support during childhood (measured with MSPSS, *r* (137) = −0.22, *p* = 0.008). In terms of habitual emotion regulation, this type of pathology correlated with instrumental goals (*r* (137) = 0.32, *p* ≤ 0.001), reappraisal (*r* (137) = −0.22, *p* = 0.007), suppression (*r* (137) = 0.34, *p* ≤ 0.001), and selective attention (*r* (137) = 0.20, *p* = 0.017). No other significant correlations were found in the case of this sensitivity analysis.

#### 3.6.2 DPD

DPD correlated negatively with OCPD (*r* (137) = −0.28, *p* ≤ 0.001). Significant associations were found between DPD and LPFS subscales: individual (*r* (137) = 0.59, *p* ≤ 0.001), interpersonal (*r* (137) = 0.30, *p* ≤ 0.001), and overall personality dysfunction (*r* (137) = 0.52, *p* ≤ 0.001). Instrumental goals positively correlated with DPD (*r* (137) = 0.35, *p* ≤ 0.001), while reappraisal negatively correlated with this personality pathology (*r* (137) = −0.22, *p* = 0.009). No other significant correlations were found in the case of this sensitivity analysis.

#### 3.6.3 OCPD

OCPD positively correlated with situation selection (*r* (137) = 0.19, *p* = 0.019). No other significant correlations were found in the case of this sensitivity analysis.

#### 3.6.4 Childhood adversity

This analysis also indicated that the measured types of childhood adversity correlated significantly with the subscales of the LPFS (see Supplementary Table S3). Non-violent discipline (measured with CTSPC) was significantly correlated with hedonic goals (*r* (137) = 0.20, *p* = 0.016) and selective attention (*r* (137) = 0.20, *p* = 0.018). Reappraisal correlated with childhood maltreatment (measured with CTQ, *r* (137) = −0.19, *p* = 0.020), perceived social support during childhood (measured with MSPSS, *r* (137) = 0.20, *p* = 0.014), psychological aggression (measured with CTSPC, *r* (137) = −0.24, *p* = 0.004), and childhood family risk (measured with RFQ, *r* (137) = −0.22, *p* = 0.008). Suppression was associated with childhood unpredictability (measured with QUIC, *r* (137) = 0.18, *p* = 0.033) and perceived social support during childhood (measured with MSPSS, *r* (137) = −0.22, *p* = 0.009).

#### 3.6.5 Habitual emotion regulation

Individual personality dysfunction was significantly correlated with hedonic goals (*r* (137) = 0.21, *p* = 0.013), instrumental goals (*r* (137) = 0.25, *p* = 0.003), reappraisal (*r* (137) = −0.28, *p* ≤ 0.001), suppression (*r* (137) = 0.26, *p* = 0.002), and selective attention (*r* (137) = 0.19, *p* = 0.020). Interpersonal personality dysfunction was associated with hedonic goals (*r* (137) = 0.18, *p* = 0.032), suppression (*r* (137) = 0.33, *p* ≤ 0.001), distraction (*r* (137) = 0.19, *p* = 0.020), and selective attention (*r* (137) = 0.32, *p* ≤ 0.001). Overall personality dysfunction was associated with hedonic goals (*r* (137) = 0.22, *p* = 0.008), instrumental goals (*r* (137) = 0.20, *p* = 0.014), reappraisal (*r* (137) = −0.22, *p* = 0.009), suppression (*r* (137) = 0.33, *p* ≤ 0.001), and selective attention (*r* (137) = 0.28, *p* ≤ 0.001). We also found positive associations between hedonic goals, suppression (*r* (137) = 0.16, *p* = 0.047), selective attention (*r* (137) = 0.21, *p* = 0.013), and situation selection (*r* (137) = 0.28, *p* ≤ 0.001). Instrumental goals were associated with suppression (*r* (137) = 0.27, *p* = 0.001), distraction (*r* (137) = 0.31, *p* ≤ 0.001), selective attention (*r* (137) = 0.26, *p* = 0.002), and situation selection (*r* (137) = 0.23, *p* = 0.006).

### 3.7 Partial correlations in the female sample

Partial correlation analysis indicated that participant age correlated individual personality dysfunction (*r* (123) = −0.23, *p* = 0.009), childhood unpredictability (measured with QUIC, *r* (123) = −0.18, *p* = 0.041), and non-violent discipline (measured with CTSPC, *r* (123) = 0.23, *p* = 0.009).

#### 3.7.1 AVPD

AVPD correlated positively with DPD (*r* (122) = 0.51, *p* ≤ 0.001), and negatively with OCPD (*r* (122) = −0.27, *p* = 0.002). Significant associations were found between AVPD and LPFS subscales: individual (*r* (122) = 0.47, *p* ≤ 0.001), interpersonal (*r* (122) = 0.43, *p* ≤ 0.001), and overall personality dysfunction (*r* (122) = 0.52, *p* ≤ 0.001). This type of pathology also correlated with perceived social support during childhood (measured with MSPSS, *r* (122) = −0.18, *p* = 0.039). In terms of habitual emotion regulation, this type of pathology correlated with instrumental goals (*r* (122) = 0.28, *p* = 0.001), reappraisal (*r* (122) = −0.28, *p* = 0.001), and suppression (*r* (122) = 0.28, *p* = 0.001). No other significant correlations were found in the case of this sensitivity analysis.

#### 3.7.2 DPD

DPD correlated negatively with OCPD (*r* (122) = −0.24, *p* = 0.005). Significant associations were found between DPD and LPFS subscales: individual (*r* (122) = 0.55, *p* ≤ 0.001), interpersonal (*r* (122) = 0.26, *p* = 0.003), and overall personality dysfunction (*r* (122) = 0.49, *p* ≤ 0.001). Instrumental goals positively correlated with DPD (*r* (122) = 0.31, *p* ≤ 0.001), while reappraisal negatively correlated with this personality pathology (*r* (122) = −0.23, *p* = 0.010). No other significant correlations were found in the case of this sensitivity analysis.

#### 3.7.3 OCPD

OCPD correlated positively with non-violent discipline (measured with CTSPC, *r* (122) = 0.18, *p* = 0.038) and situation selection (*r* (122) = 0.18, *p* = 0.041). No other significant correlations were found in the case of this sensitivity analysis.

#### 3.7.4 Childhood adversity

This analysis also indicated that the measured types of childhood adversity correlated significantly with the subscales of the LPFS (see Supplementary Table S10). Non-violent discipline (measured with CTSPC) was significantly correlated with hedonic goals (*r* (122) = 0.18, *p* = 0.042). Reappraisal correlated with childhood maltreatment (measured with CTQ, *r* (122) = −0.25, *p* = 0.005), perceived social support during childhood (measured with MSPSS, *r* (122) = 0.24, *p* = 0.006), psychological aggression (measured with CTSPC, *r* (122) = −0.31, *p* ≤ 0.001), and childhood family risk (measured with RFQ, *r* (122) = −0.29, *p* = 0.001).

#### 3.7.5 Habitual emotion regulation

Individual personality dysfunction was significantly correlated with hedonic goals (*r* (122) = 0.18, *p* = 0.040), instrumental goals (*r* (122) = 0.21, *p* = 0.017), reappraisal (*r* (122) = −0.30, *p* ≤ 0.001), suppression (*r* (122) = 0.24, *p* = 0.006), and selective attention (*r* (122) = 0.21, *p* = 0.019). Interpersonal personality dysfunction was associated with suppression (*r* (122) = 0.31, *p* ≤ 0.001), and selective attention (*r* (122) = 0.29, *p* = 0.001). Overall personality dysfunction was associated with hedonic goals (*r* (122) = 0.20, *p* = 0.021), reappraisal (*r* (122) = −0.25, *p* = 0.005), suppression (*r* (122) = 0.31, *p* ≤ 0.001), and selective attention (*r* (122) = 0.28, *p* = 0.001). We also found positive associations between hedonic goals, selective attention (*r* (122) = 0.19, *p* = 0.028), and situation selection (*r* (122) = 0.31, *p* ≤ 0.001). Instrumental goals were associated with suppression (*r* (122) = 0.23, *p* = 0.010), distraction (*r* (122) = 0.26, *p* = 0.003), selective attention (*r* (122) = 0.26, *p* = 0.004), and situation selection (*r* (122) = 0.25, *p* = 0.005).

### 3.8 Moderation results

#### 3.8.1 Main analysis using AVPD as the outcome

The overall models where childhood maltreatment (measured using the CTQ) was used as a moderator of the associations between hedonic goals and AVPD (*R*^2^= 0.010, *F*_(3, 137)_ = 0.49, *p* = 0.688), reappraisal and AVPD (*R*^2^ = 0.053, *F*_(3, 137)_ = 2.59, *p* = 0.054), distraction and AVPD (*R*^2^ = 0.030, *F*_(3, 137)_ = 1.41, *p* = 0.241), and situation selection and AVPD (*R*^2^ = 0.039, *F*_(3, 137)_ = 1.86, *p* = 0.139) were not significant.

The overall model using childhood maltreatment as a moderator for the associations between instrumental goals and AVPD, (*R*^2^ = 0.123, *F*_(3, 137)_ = 6.41, *p* < 0.001), between suppression and AVPD (*R*^2^ = 0.116, *F*_(3, 137)_ = 6.02, *p* < 0.001), and between selective attention and AVPD (*R*^2^ = 0.058, *F*_(3, 137)_ = 2.85, *p* = 0.039) were significant. However, childhood maltreatment did not moderate any of the investigated relationships (see Table 4).

**Table 4:** Moderation results for avoidant personality disorder (*N* = 141).

Factor	*b*	SE	*t*	*p*	95% CI	
					LL	UL
Hedonic goals	−0.203	1.537	−0.132	0.895	−3.242	2.836
CTQ	−0.0001	0.106	−0.001	0.998	−0.210	0.210
Hedonic goals^*^CTQ	0.006	0.026	0.230	0.817	−0.046	0.058
Instrumental goals	2.298^*^	1.078	2.130	0.034	0.165	4.431
CTQ	0.100	0.107	0.932	0.353	−0.112	0.313
Instrumental goals^*^CTQ	−0.015	0.020	−0.775	0.439	−0.055	0.024
Reappraisal	−0.504	0.976	−0.516	0.606	−2.434	1.426
CTQ	0.036	0.085	0.434	0.664	−0.131	0.205
Reappraisal^*^CTQ	−0.004	0.017	−0.278	0.781	−0.038	0.028
Suppression	1.380	0.793	1.740	0.084	−0.187	2.949
CTQ	0.036	0.067	0.543	0.587	−0.097	0.170
Suppression^*^CTQ	−0.005	0.014	−0.361	0.718	−0.034	0.023
Distraction	0.612	0.985	0.621	0.535	−1.336	2.561
CTQ	0.033	0.088	0.380	0.704	−0.141	0.209
Distraction^*^CTQ	−0.002	0.017	−0.118	0.906	−0.037	0.033
Selective attention	1.332	0.765	1.740	0.084	−0.181	2.845
CTQ	0.072	0.054	1.325	0.187	−0.035	0.181
Selective attention^*^CTQ	−0.012	0.013	−0.903	0.367	−0.039	0.014
Situation selection	0.640	0.834	0.767	0.443	−1.009	2.290
CTQ	0.115	0.073	1.565	0.119	−0.030	0.261
Situation selection^*^CTQ	−0.020	0.015	−1.333	0.184	−0.049	0.009

^*^p ≤ 0.05. CTQ, Childhood maltreatment.

#### 3.8.2 Sensitivity analyses using AVPD as the outcome

The models where childhood maltreatment was employed as a moderator in the associations between hedonic goals and AVPD (*R*^2^= 0.045, *F*_(5, 135)_ = 0.76, *p* = 0.576), distraction and AVPD (*R*^2^ = 0.058, *F*_(5, 135)_ = 1.21, *p* = 0.306), selective attention and AVPD (*R*^2^ = 0.087, *F*_(5, 135)_ = 2.02, *p* = 0.079) were not significant. However, we found significant models in the case of the associations between instrumental goals and AVPD, (*R*^2^ = 0.142, *F*_(5, 135)_ = 3.53, *p* = 0.004), reappraisal and AVPD (*R*^2^ = 0.086, *F*_(5, 135)_ = 2.95, *p* = 0.014), suppression and AVPD (*R*^2^ = 0.151, *F*_(5, 135)_ = 3.95, *p* = 0.002), situation selection and AVPD (*R*^2^ = 0.071, *F*_(5, 135)_ = 2.44, *p* = 0.037). The paths between variables, controlling for age and sex are presented in Supplementary Table S7. Childhood maltreatment did not moderate any of the investigated paths.

#### 3.8.3 Sensitivity analyses in the female sample using AVPD as the outcome

In the case of the female sample, the overall models of hedonic goals and AVPD (*R*^2^= 0.018, *F*_(4, 120)_ = 0.40, *p* = 0.807), distraction and AVPD (*R*^2^ = 0.023, *F*_(4, 120)_ = 0.54, *p* = 0.701), selective attention and AVPD (*R*^2^ = 0.051, *F*_(4, 120)_ = 1.29, *p* = 0.274), situation selection and AVPD (*R*^2^ = 0.052, *F*_(4, 120)_ = 2.26, *p* = 0.066) were not significant. The overall models of the pathways between instrumental goals and AVPD, (*R*^2^ = 0.099, *F*_(4, 120)_ = 2.77, *p* = 0.030), reappraisal and AVPD (*R*^2^ = 0.100, *F*_(4, 120)_ = 3.35, *p* = 0.012), and suppression and AVPD (*R*^2^ = 0.096, *F*_(4, 120)_ = 2.84, *p* = 0.027) were significant, but childhood maltreatment did not moderate any of these associations (see Supplementary Table S11).

#### 3.8.4 Main analysis using DPD as the outcome

The overall models indicated no significant impact of childhood maltreatment (measured using CTQ) on associations with DPD for the following independent variables: hedonic goals (*R*^2^ = 0.009, *F*_(3, 137)_ = 0.44, *p* = 0.722), reappraisal (*R*^2^ = 0.050, *F*_(3, 137)_ = 2.42, *p* = 0.068), suppression (*R*^2^ = 0.025, *F*_(3, 137)_ = 1.21, *p* = 0.307), distraction (*R*^2^ = 0.017, *F*_(3, 137)_ = 0.81, *p* = 0.485), selective attention, (*R*^2^ = 0.032, *F*_(3, 137)_ = 1.54, *p* = 0.205), and situation selection, (*R*^2^ = 0.035, *F*_(3, 137)_ = 1.68, *p* = 0.173). A significant overall effect of childhood maltreatment was identified on the association between instrumental goals and DPD, *R*^2^ = 0.118, *F*_(3, 137)_ = 6.12, *p* < 0.001, but childhood maltreatment did not moderate any of the investigated associations (see Table 5).

**Table 5:** Moderation results for dependent personality disorder (*N* = 141).

Factor	*b*	SE	*t*	*p*	95% CI	
					LL	UL
Hedonic goals	0.087	1.770	0.049	0.960	−3.413	3.588
CTQ	−0.002	0.122	−0.021	0.983	−0.244	0.239
Hedonic goals^*^CTQ	0.005	0.030	0.192	0.847	−0.054	0.065
Instrumental goals	2.180	1.245	1.750	0.082	−0.282	4.642
CTQ	0.058	0.124	0.468	0.640	−0.187	0.304
Instrumental goals^*^CTQ	−0.008	0.023	−0.360	0.719	−0.054	0.037
Reappraisal	−1.327	1.125	−1.179	0.240	−3.554	0.898
CTQ	−0.030	0.098	−0.313	0.754	−0.224	0.163
Reappraisal^*^CTQ	0.008	0.019	0.420	0.675	−0.030	0.047
Suppression	1.062	0.958	1.108	0.269	−0.833	2.957
CTQ	0.062	0.081	0.764	0.445	−0.099	0.224
Suppression^*^CTQ	−0.010	0.017	−0.597	0.551	−0.045	0.024
Distraction	0.357	1.141	0.313	0.754	−1.900	2.615
CTQ	0.012	0.102	0.119	0.905	−0.190	0.215
Distraction^*^CTQ	0.001	0.020	0.081	0.935	−0.039	0.042
Selective attention	0.632	0.893	0.707	0.480	−1.134	2.398
CTQ	0.023	0.064	0.370	0.711	−0.103	0.150
Selective attention^*^CTQ	−0.0001	0.015	−0.009	0.992	−0.031	0.031
Situation selection	1.312	0.962	1.364	0.174	−0.590	3.215
CTQ	0.164	0.085	1.934	0.055	−0.003	0.333
Situation selection^*^CTQ	−0.031	0.017	−1.787	0.076	−0.065	0.003

CTQ, Childhood maltreatment.

#### 3.8.5 Sensitivity analyses using DPD as the outcome

The models where childhood maltreatment was employed as a moderator in the associations between hedonic goals and DPD (*R*^2^= 0.018, *F*_(5, 135)_ = 0.46, *p* = 0.804), reappraisal and DPD (*R*^2^ = 0.059, *F*_(5, 135)_ = 1.88, *p* = 0.100), suppression and DPD (*R*^2^ = 0.035, *F*_(5, 135)_ = 0.84, *p* = 0.518), distraction and DPD (*R*^2^ = 0.029, *F*_(5, 135)_ = 0.95, *p* = 0.450), selective attention and DPD (*R*^2^ = 0.043, *F*_(5, 135)_ = 1.16, *p* = 0.331), situation selection and DPD (*R*^2^ = 0.047, *F*_(5, 135)_ = 1.65, *p* = 0.148) were not significant. The model pertaining to instrumental goals and DPD, (*R*^2^ = 0.138, *F*_(5, 135)_ = 5.08, *p* = 0.0003) was, however, significant. The paths of this model are presented in Supplementary Table S8. Childhood maltreatment did not moderate any of the investigated associations.

#### 3.8.6 Sensitivity analyses in the female sample using DPD as the outcome

The overall models investigating whether childhood maltreatment was a moderator in the pathways between hedonic goals and DPD (*R*^2^= 0.010, *F*_(4, 120)_ = 0.26, *p* = 0.901), reappraisal and DPD (*R*^2^ = 0.063, *F*_(4, 120)_ = 2.39, *p* = 0.054), suppression and DPD (*R*^2^ = 0.027, *F*_(4, 120)_ = 0.65, *p* = 0.621), distraction and DPD (*R*^2^ = 0.014, *F*_(4, 120)_ = 0.50, *p* = 0.733), selective attention and DPD (*R*^2^ = 0.036, *F*_(4, 120)_ = 1.08, *p* = 0.367), and situation selection and DPD (*R*^2^ = 0.036, *F*_(4, 120)_ = 1.33, *p* = 0.259) were not significant. The overall model of the pathways between instrumental goals and DPD, (*R*^2^ = 0.114, *F*_(4, 120)_ = 4.77, *p* = 0.001) was significant, but childhood maltreatment did not moderate any of these associations (see Supplementary Table S12).

#### 3.8.7 Main analysis using OCPD as the outcome

**Figure 1:**
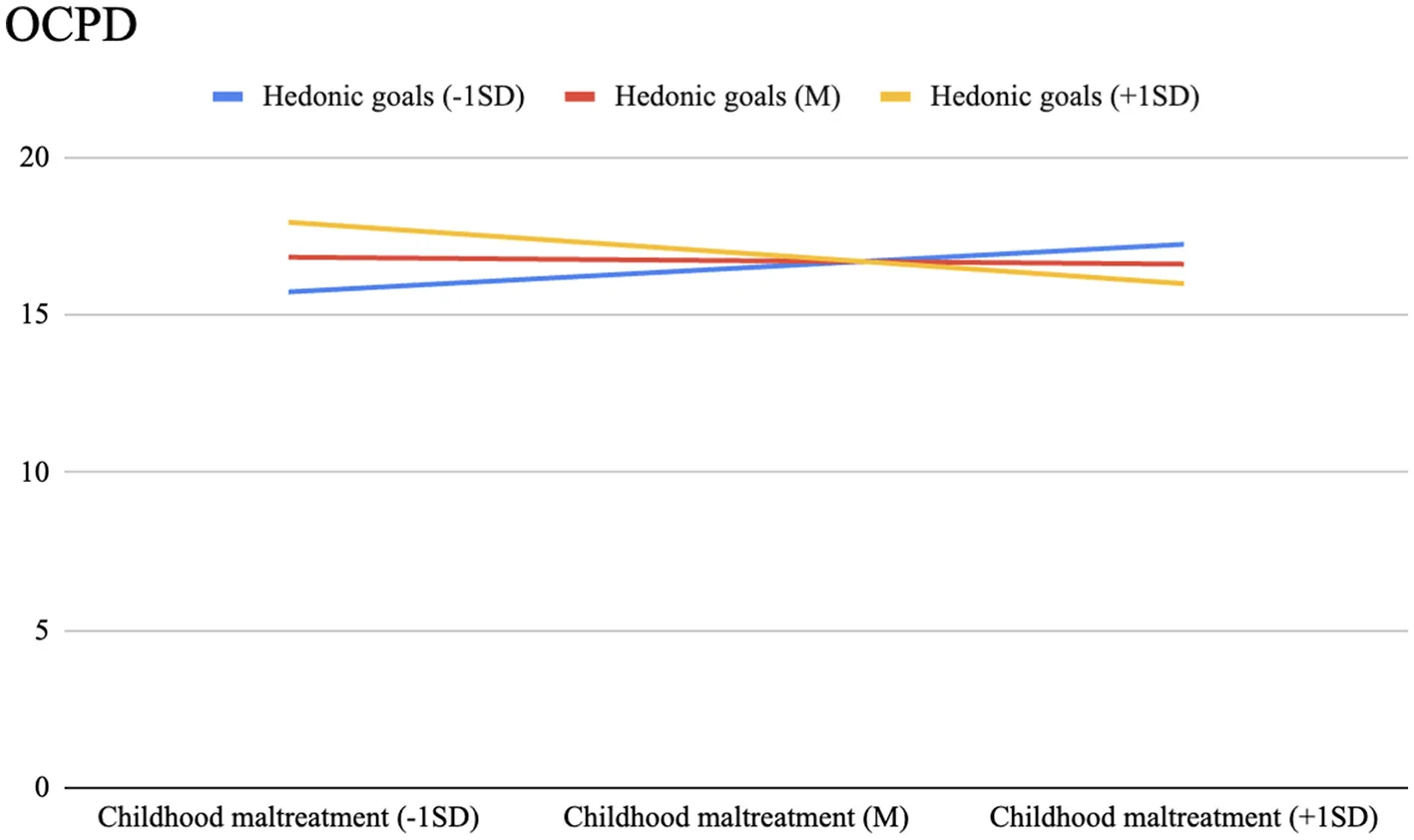
Regression plot for the association between hedonic goals and obsessive-compulsive personality disorder (OCPD), using childhood maltreatment (CTQ) as a moderator.

No significant overall models were identified for the impact of childhood maltreatment (measured using CTQ) on associations with OCPD for the following predictors: instrumental goals (*R*^2^ = 0.011, *F*_(3, 137)_ = 0.53, *p* = 0.658), reappraisal (*R*^2^ = 0.017, *F*_(3, 137)_ = 0.78, *p* = 0.501), suppression (*R*^2^ = 0.014, *F*_(3, 137)_ = 0.67, *p* = 0.568), distraction (*R*^2^ = 0.011, *F*_(3, 137)_ = 0.54, *p* = 0.651), selective attention, (*R*^2^ = 0.015, *F*_(3, 137)_ = 0.73, *p* = 0.531), and situation selection, (*R*^2^ = 0.043, *F*_(3, 137)_ = 2.08, *p* = 0.104). However, a significant overall model was identified for the impact of childhood maltreatment on the association between hedonic goals and OCPD, *R*^2^ = 0.063, *F*_(3, 137)_ = 3.11, *p* = 0.028, with results indicating that childhood maltreatment moderated this association (see Table 6). Figure 1 presents the regression plot for this analysis.

**Table 6:** Moderation results for obsessive-compulsive personality disorder (*N* = 141).

Factor	*b*	SE	*t*	*p*	95% CI	
					LL	UL
Hedonic goals	3.560^*^	1.209	2.944	0.003	1.169	5.950
CTQ	0.232^*^	0.083	2.782	0.006	0.067	0.398
Hedonic goals^*^CTQ	−0.061^*^	0.020	−2.962	0.003	−0.102	−0.020
Instrumental goals	0.131	0.926	0.141	0.887	−1.699	1.962
CTQ	0.037	0.092	0.400	0.689	−0.146	0.220
Instrumental goals^*^CTQ	−0.008	0.017	−0.506	0.613	−0.042	0.025
Reappraisal	0.865	0.804	1.076	0.283	−0.725	2.456
CTQ	0.043	0.070	0.626	0.531	−0.094	0.182
Reappraisal^*^CTQ	−0.010	0.014	−0.738	0.461	−0.038	0.017
Suppression	0.853	0.677	1.259	0.209	−0.486	2.192
CTQ	0.059	0.057	1.029	0.305	−0.054	0.173
Suppression^*^CTQ	−0.015	0.012	−1.269	0.206	−0.040	0.008
Distraction	0.839	0.804	1.043	0.298	−0.751	2.430
CTQ	0.068	0.072	0.948	0.344	−0.074	0.211
Distraction^*^CTQ	−0.016	0.014	−1.120	0.264	−0.044	0.012
Selective attention	0.849	0.632	1.341	0.182	−0.402	2.100
CTQ	0.040	0.045	0.890	0.374	−0.049	0.130
Selective attention^*^CTQ	−0.013	0.011	−1.187	0.237	−0.035	0.008
Situation selection	0.113	0.673	0.169	0.866	−1.217	1.444
CTQ	−0.041	0.059	−0.696	0.487	−0.159	0.076
Situation selection^*^CTQ	0.007	0.012	0.611	0.542	−0.016	0.031

^*^p ≤ 0.05. CTQ, Childhood maltreatment.

#### 3.8.8 Sensitivity analyses using OCPD as the outcome

Once we controlled for age and sex, none of the investigated models where childhood maltreatment moderated the associations between hedonic goals and OCPD (*R*^2^= 0.068, *F*_(5, 135)_ = 1.71, *p* = 0.134), instrumental goals and OCPD, (*R*^2^ = 0.013, *F*_(5, 135)_ = 0.32, *p* = 0.895), reappraisal and OCPD (*R*^2^ = 0.019, *F*_(5, 135)_ = 0.71, *p* = 0.611), suppression and OCPD (*R*^2^ = 0.018, *F*_(5, 135)_ = 0.49, *p* = 0.783), distraction and OCPD (*R*^2^ = 0.013, *F*_(5, 135)_ = 0.48, *p* = 0.787), selective attention and OCPD (*R*^2^ = 0.019, *F*_(5, 135)_ = 0.48, *p* = 0.785), and situation selection and OCPD (*R*^2^ = 0.044, *F*_(5, 135)_ = 1.29, *p* = 0.270) were significant (see Supplementary Table S9). The interaction of hedonic goals^*^CTQ remained significant, suggesting a moderating effect of childhood maltreatment in the association between hedonic goals and OCPD (see Supplementary Table S9).

#### 3.8.9 Sensitivity analyses in the female sample using OCPD as the outcome

While the overall models for the pathway between hedonic goals and OCPD (*R*^2^= 0.074, *F*_(4, 120)_ = 2.52, *p* = 0.044) was significant, the overall models for instrumental goals and OCPD, (*R*^2^ = 0.010, *F*_(4, 120)_ = 0.29, *p* = 0.881), reappraisal and OCPD (*R*^2^ = 0.026, *F*_(4, 120)_ = 1.09, *p* = 0.361), suppression and OCPD (*R*^2^ = 0.016, *F*_(4, 120)_ = 0.56, *p* = 0.692), distraction and OCPD (*R*^2^ = 0.015, *F*_(4, 120)_ = 0.58, *p* = 0.676), selective attention and OCPD (*R*^2^ = 0.021, *F*_(4, 120)_ = 0.62, *p* = 0.648), and situation selection and OCPD (*R*^2^ = 0.038, *F*_(4, 120)_ = 1.34, *p* = 0.258) were not significant (see Supplementary Table S13). The interaction of hedonic goals^*^CTQ remained significant, suggesting a moderating effect of childhood maltreatment in the association between hedonic goals and OCPD. (see Supplementary Table S13).

In the main analyses, the hypothesis that childhood maltreatment moderates the associations between habitual emotion regulation, AVPD and DPD, was invalidated. In the case of OCPD, the hypothesis was confirmed only in the case of the pathway between hedonic goals and OCPD. This effect remained constant in the conducted sensitivity analyses.

## 4 Discussion

This study set out to investigate the associations between facets of childhood adversity, habitual emotion regulation and cluster C personality disorder symptoms. We tested whether childhood maltreatment moderated the relationships between habitual emotion regulation goals and strategies, AVPD, DPD, and OCPD symptoms. The findings of this study provide partial support for the hypothesized associations and bring forward empirical data on emotion regulation goals in individuals presenting cluster C symptomatology, which were previously investigated or theorized solely in the case of borderline PD (López-Pérez and McCagh, 2020; Millgram et al., 2020).

We found significant associations between AVPD, DPD, and OCPD. As AVPD and DPD symptomatology increased, OCPD symptomatology decreased. AVPD and DPD symptomatology was robustly associated with higher impairments in personality functioning, while OCPD showed no significant associations with personality dysfunction. These results are consistent with previous observations that these disorders differ, even though they are included in the same personality disorders cluster (De Reus and Emmelkamp, 2012; Reichborn-Kjennerud et al., 2007). These findings also support the view that individuals with OCPD tend to rate themselves as high functioning when compared with individuals presenting other personality disorders (De Reus and Emmelkamp, 2012; Massaal-van der Ree et al., 2022; Pinto et al., 2007; Skodol et al., 2002).

As childhood unpredictability increased, and perceived social support decreased, AVPD symptomatology increased. In the female sample, non-violent discipline was associated with higher levels of OCPD symptomatology. These results are in line with previous literature suggesting that childhood adversity is associated with vulnerability for AVPD symptomatology (Beck et al., 2015; Gunay-Oge et al., 2020; Johnson et al., 2006) and with OCPD traits (Gray, 2023; Gray et al., 2024). No other significant associations were found between cluster C and childhood adversity. There are some studies that did not identify significant associations between these constructs (e.g., DeJong et al., 1995; Mertens et al., 2020; Zhang and Zheng, 2018). These results could be due to the use of retrospective, self-report instruments employed to assess childhood adversity, as ACEs tend to be underreported in these types of instruments, which could undermine the investigated associations (Finkelhor, 2018; Thabrew et al., 2012). In contrast, several forms of childhood adversity were associated with higher personality dysfunction, supporting the view that exposure to adversities during childhood is associated with poor personality functioning in the case of cluster C (Chiesa et al., 2016; Massaal-van der Ree et al., 2022).

Turning to emotion regulation, both AVPD and DPD symptoms were positively associated with instrumental goals and negatively associated with reappraisal. AVPD was additionally linked to higher usage of suppression and selective attention, while OCPD was associated with greater usage of situation selection. Habitual hedonic and instrumental goals, lower usage of reappraisal and higher usage of suppression, distraction, and selective attention were all associated with increases in personality dysfunction. These findings extend previous reports of emotion regulation difficulties in the case of cluster C (e.g., Borges and Naugle, 2017; Snir et al., 2017), by showing that individuals with AVPD and DPD symptoms appear more oriented toward instrumental motives (i.e., performance, impression management, or relationship maintenance) and less inclined to use reappraisal. The associations found between AVPD, suppression and selective attention, and between OCPD with situation selection, are compatible with the experiential avoidance and over-control described in previous studies (Lampe, 2016; Wheaton and Pinto, 2017), considering the literature suggests that the use of strategies such as distraction, selective attention, and situation selection entail disengagement from emotional experiences (Andriopoulos and Kafetsios, 2015; Gross et al., 2019; McMahon and Naragon-Gainey, 2019). Hedonic and instrumental goals were related to most regulation strategies (except reappraisal), supporting the view that regulation goals contribute to strategy selection (Eldesouky and English, 2019b; Tamir, 2016).

Several facets of childhood adversity were associated with habitual emotion regulation. Higher levels of childhood maltreatment, psychological aggression, and family risk were linked to lower reappraisal, while greater levels of childhood maltreatment and childhood unpredictability were associated with higher usage of suppression. Lower perceived social support was related to both lower reappraisal and higher suppression. Non-violent discipline showed positive associations with hedonic goals and selective attention. These patterns are consistent with prior evidence that adverse childhood experiences are related to reduced use of adaptive strategies such as reappraisal and increased reliance on less adaptive strategies such as suppression (Ion et al., 2023; Miu et al., 2022; Lavi et al., 2019).

In the main analysis, childhood maltreatment moderated only the association between hedonic goals and OCPD symptoms. Higher levels of both childhood maltreatment and hedonic goals were linked to lower OCPD symptomatology. All the other tested interactions were non-significant. Importantly, this single interaction remained consistent in the sensitivity analyses that controlled for age and sex or that restricted the sample to women. Thus, the hypothesis that pertains to childhood maltreatment moderating the associations between habitual emotion regulation and AVPD, DPD, and OCPD receives very limited support. One possible interpretation of this finding is that, among individuals with higher childhood maltreatment, a stronger orientation toward hedonic goals may temporarily loosen the rigid emotional over-control that characterizes OCPD. The overall pattern identified in this study suggests that childhood maltreatment does not systematically alter the links between habitual emotion regulation and cluster C symptoms in this community sample. Because of the exploratory nature of these analyses and the number of tests conducted, this finding should be regarded as preliminary.

The present study expands current literature by taking into account extensive aspects of childhood adversity, from a continuous, dimensional perspective, as has been underlined and suggested in previous literature (Finkelhor et al., 2015; Glynn et al., 2019; McLaughlin and Sheridan, 2016; Smith and Pollak, 2021). Given that these extended facets of adversity have not yet been investigated in the case of cluster C, this study could have important contributions to the understanding of etiological factors involved in these disorders. Furthermore, this study adds to the literature by investigating novel emotion regulation processes (i.e., goals) in the case of cluster C.

Emotion regulation has received less attention in preceding research in the case of cluster C personality disorders. Further investigation into the processes of emotion regulation (e.g., the selection and implementation stages of emotion regulation) and their role in the course and development of cluster C personality disorders is necessary. Uncovering the interaction between childhood adversity and emotion regulation in the case of cluster C could lead to a better understanding of this symptomatology and to the development of more effective prevention and intervention efforts. So far, this study provides further evidence of the presence of emotion regulation difficulties in the case of cluster C, by investigating possible emotion regulation goals that generally motivate regulatory efforts in individuals presenting this type of symptomatology and by underlining their possible contribution to OCPD symptomatology.

### 4.1 Limitations and future directions

This study employs cross-sectional, self-reported data. Previous studies suggest that this type of data could be subjected to biases (Rosenman et al., 2011; Solem, 2015; Woodside, 2011), especially when assessing constructs such as childhood adversity (Smith and Pollak, 2021) or emotion regulation (Ion et al., 2023). In order to draw firmer conclusions, studies that investigate these associations using prospective, longitudinal, experimental or ecological measures are necessary. These types of designs would allow to assess how these processes and their interactions unfold over time, across different contexts. Some results of this study should be interpreted with caution, as some scales presented low Cronbach α coefficients.

Participants included in the study were extracted from the community, and the sample consisted mostly of women. We recruited the sample via social media advertisements (i.e., Facebook and Instagram). Prior research indicates that this recruitment method frequently yields samples with an overrepresentation of young women (Whitaker et al., 2017). This is likely to have contributed to the marked sex imbalance observed in the present study. To mitigate this concern, we conducted sensitivity analyses that controlled for sex and repeated the main analyses in the female-only subsample (*N* = 125). The patterns of the present results remained largely consistent, but the generalizability of the findings to male populations is limited. A further limitation concerns the exploratory status of the moderation analyses. We tested a relatively large number of models (seven emotion regulation predictors across three Cluster C outcomes). Given the number of tests performed and the absence of a formal multiple-comparison correction, this single finding must be interpreted cautiously. It should be viewed as hypothesis-generating rather than confirmatory and awaits replication in independent samples before strong theoretical or clinical conclusions are drawn.

Even though we measured personality impairment, these dysfunctions were not taken into account when measuring cluster C personality disorder symptoms, nor did we diagnose participants using clinical interviews. Moreover, although the presence of other personality disorder symptoms was measured, the presence of comorbidities was not taken into account. The sample consisted mostly of adult women. Age and sex differences could not be investigated. Consequently, the results of this study can be generalized to individuals presenting mild to moderate severity of cluster C symptomatology. Even though samples extracted from the community present some advantages (Acharya et al., 2013), these results also need to be replicated in the case of participants presenting clinical cluster C personality disorders (which typically present increased comorbidity with other personality disorders and clinical conditions) (e.g., Disney, 2013; Weinbrecht et al., 2016), using clinical interviews when conducting diagnosis. Taking functional impairment and comorbidities into consideration when investigating cluster C would also be important, to get a better understanding of these disorders.

This study concentrated on broader categories of emotion regulation goals (i.e., hedonic and instrumental goals). Future studies could look further into these goals. For example, studies could try to disentangle whether individuals with cluster C symptoms are generally motivated to increase or reduce emotional distress. Moreover, we looked at emotion regulation strategies as specified by the emotion regulation process model (Gross, 1998, 2015). Future studies could investigate other emotion regulation strategies, such as rumination or experiential avoidance, and their role in the association between childhood adversity and cluster C.

We have investigated broader aspects of childhood adversity. However, more research is necessary into their association with cluster C personality disorders. Future studies could investigate whether developmental features of childhood adversity (i.e., intensity, frequency, chronicity of childhood adversity) (Dunn et al., 2018; Smith and Pollak, 2021) influence these associations. Prospective measures are also necessary in order to better understand these relationships and their dynamics. Further investigation is also necessary into the mechanisms that explain the associations between childhood adversity and cluster C personality disorders. Studies could extend research on regulatory processes in the case of these disorders and could investigate how cognitive mechanisms (such as early maladaptive schemas) (Pilkington et al., 2021) fare in the associations between childhood adversity and this type of symptomatology, especially when taking emotion regulation into account.

## Data Availability

The raw data supporting the conclusions of this article will be made available by the authors, without undue reservation. Requests to access the datasets should be directed to the first author.
