# Supplementary material for: The association between habitual emotion regulation, childhood adversity, and cluster C personality disorders symptomatology
Source: Front Psychol. 2026 Sep 3;17:1893753. doi: 10.3389/fpsyg.2026.1893753 (PMC13588754; doi:10.3389/fpsyg.2026.1893753)
Supplement: Supplementary file 1 [file Data_Sheet_1.docx]

**Supplementary material 1**

1. **Assumption checks for correlation**
   1.1. **Normality**

**Table S1.** Shapiro-Wilk test results

| Variable | Statistic | df | *p* |
| --- | --- | --- | --- |
| SNAP – AVPD | .866** | 141 | < .001 |
| SNAP – DPD | .957** | 141 | < .001 |
| SNAP – OCPD | .959** | 141 | < .001 |
| LPFS - Individual personality dysfunction | .961** | 141 | < .001 |
| LPFS - Interpersonal personality dysfunction | .971* | 141 | .005 |
| LPFS - Overall personality dysfunction | .992 | 141 | .609 |
| Socio-economic status during childhood | .969* | 141 | .003 |
| CTQ - Childhood maltreatment | .957** | 141 | < .001 |
| QUIC - Childhood unpredictability | .965** | 141 | .001 |
| MSPSS - Childhood perceived social support | .950** | 141 | < .001 |
| CTSPC - Non-violent discipline | .986 | 141 | .183 |
| CTSPC - Psychological aggression | .959** | 141 | < .001 |
| CTSPC - Physical assault | .930** | 141 | < .001 |
| CTSPC - Neglect | .952** | 141 | < .001 |
| CTSPC - Sexual abuse | .653** | 141 | < .001 |
| RFQ - Childhood family risk | .971* | 141 | .005 |
| ERGS - Hedonic goals | .986 | 141 | .172 |
| ERGS - Instrumental goals | .979* | 141 | .027 |
| E-ERQ - Reappraisal | .977* | 141 | .016 |
| E-ERQ - Suppression | .987 | 141 | .184 |
| E-ERQ - Distraction | .980* | 141 | .034 |
| E-ERQ - Selective attention | .986 | 141 | .170 |
| E-ERQ - Situation selection | .967* | 141 | .002 |

**p ≤ .05; **p ≤ .001.*

**Figure S1.** Histograms

**Figure S1.** Histograms *(continued)*

**Figure S1.** Histograms *(continued)*

**Figure S1.** Histograms *(continued)*

*Note.* Histograms showing the frequency distribution of: A = Avoidant PD; B = Dependent PD; C = Obsessive-compulsive PD; D = LPFS - Individual personality dysfunction; E = LPFS - Interpersonal personality dysfunction; F = LPFS - Overall personality dysfunction; G = Socio-economic status during childhood; H = CTQ - Childhood maltreatment; I = QUIC - Childhood unpredictability; J = MSPSS - Childhood perceived social support; K = CTSPC - Non-violent discipline; L = CTSPC - Psychological aggression; M = CTSPC - Physical assault; N = CTSPC – Neglect; O = CTSPC - Sexual abuse; P = RFQ - Childhood family risk; Q = ERGS - Hedonic goals; R = ERGS - Instrumental goals; S = E-ERQ – Reappraisal; T = E-ERQ – Suppression; U = E-ERQ – Distraction; V = E-ERQ - Selective attention; W = E-ERQ - Situation selection.

**Figure S2.** Normal Q-Q plots

**Figure S2.** Normal Q-Q plots *(continued)*

**Figure S2.** Normal Q-Q plots *(continued)*

**Figure S2.** Normal Q-Q plots *(continued)*

*Note.* Normal Q-Q plots showing the normal distribution of: A = Avoidant PD; B = Dependent PD; C = Obsessive-compulsive PD; D = LPFS - Individual personality dysfunction; E = LPFS - Interpersonal personality dysfunction; F = LPFS - Overall personality dysfunction; G = Socio-economic status during childhood; H = CTQ - Childhood maltreatment; I = QUIC - Childhood unpredictability; J = MSPSS - Childhood perceived social support; K = CTSPC - Non-violent discipline; L = CTSPC - Psychological aggression; M = CTSPC - Physical assault; N = CTSPC – Neglect; O = CTSPC - Sexual abuse; P = RFQ - Childhood family risk; Q = ERGS - Hedonic goals; R = ERGS - Instrumental goals; S = E-ERQ – Reappraisal; T = E-ERQ – Suppression; U = E-ERQ – Distraction; V = E-ERQ - Selective attention; W = E-ERQ - Situation selection.
