# Supplementary material for: The association between habitual emotion regulation, childhood adversity, and cluster C personality disorders symptomatology
Source: Front Psychol. 2026 Sep 3;17:1893753. doi: 10.3389/fpsyg.2026.1893753 (PMC13588754; doi:10.3389/fpsyg.2026.1893753)
Supplement: Supplementary file 2 [file Data_Sheet_2.docx]

**Supplementary material 2**

1.2. **Presence of outliers**

**Figure S3.** Boxplots

**Figure S3.** Boxplots *(continued)*

**Figure S3.** Boxplots *(continued)*

**Figure S3.** Boxplots *(continued)*

*Note.* Boxplots showing the presence of outliers for: A = Avoidant PD; B = Dependent PD; C = Obsessive-compulsive PD; D = LPFS - Individual personality dysfunction; E = LPFS - Interpersonal personality dysfunction; F = LPFS - Overall personality dysfunction; G = Socio-economic status during childhood; H = CTQ - Childhood maltreatment; I = QUIC - Childhood unpredictability; J = MSPSS - Childhood perceived social support; K = CTSPC - Non-violent discipline; L = CTSPC - Psychological aggression; M = CTSPC - Physical assault; N = CTSPC – Neglect; O = CTSPC - Sexual abuse; P = RFQ - Childhood family risk; Q = ERGS - Hedonic goals; R = ERGS - Instrumental goals; S = E-ERQ – Reappraisal; T = E-ERQ – Suppression; U = E-ERQ – Distraction; V = E-ERQ - Selective attention; W = E-ERQ - Situation selection.
