# Supplementary material for: The association between habitual emotion regulation, childhood adversity, and cluster C personality disorders symptomatology
Source: Front Psychol. 2026 Sep 3;17:1893753. doi: 10.3389/fpsyg.2026.1893753 (PMC13588754; doi:10.3389/fpsyg.2026.1893753)
Supplement: Supplementary file 3 [file Data_Sheet_3.docx]

**Supplementary material 3**

1.3. **Linearity and Homogeneity of variances**

**Figure S4.** Scatterplot using Avoidant PD as the dependent variable

**Figure S4.** Scatterplot using Avoidant PD as the dependent variable *(continued)*

**Figure S4.** Scatterplot using Avoidant PD as the dependent variable *(continued)*

**Figure S4.** Scatterplot using Avoidant PD as the dependent variable *(continued)*

*Note*. Scatterplots showing the distribution of the correlations between Avoidant PD and: A = Dependent PD; B = Obsessive-compulsive PD; C = LPFS - Individual personality dysfunction; D = LPFS - Interpersonal personality dysfunction; E = LPFS - Overall personality dysfunction; F = Socio-economic status during childhood; G = CTQ - Childhood maltreatment; H = QUIC - Childhood unpredictability; I = MSPSS - Childhood perceived social support; J = CTSPC - Non-violent discipline; K = CTSPC - Psychological aggression; L = CTSPC - Physical assault; M = CTSPC – Neglect; N = CTSPC - Sexual abuse; O = RFQ - Childhood family risk; P = ERGS - Hedonic goals; Q = ERGS - Instrumental goals; R = E-ERQ – Reappraisal; S = E-ERQ – Suppression; T = E-ERQ – Distraction; U = E-ERQ - Selective attention; V = E-ERQ - Situation selection.

**Figure S5.** Scatterplot using Dependent PD as the dependent variable

**Figure S5.** Scatterplot using Dependent PD as the dependent variable *(continued)*

**Figure S5.** Scatterplot using Dependent PD as the dependent variable *(continued)*

**Figure S5.** Scatterplot using Dependent PD as the dependent variable *(continued)*

*Note*. Scatterplots showing the distribution of the correlations between Dependent PD and: A = Obsessive-compulsive PD; B = LPFS - Individual personality dysfunction; C = LPFS - Interpersonal personality dysfunction; D = LPFS - Overall personality dysfunction; E = Socio-economic status during childhood; F = CTQ - Childhood maltreatment; G = QUIC - Childhood unpredictability; H = MSPSS - Childhood perceived social support; I = CTSPC - Non-violent discipline; J = CTSPC - Psychological aggression; K = CTSPC - Physical assault; L = CTSPC – Neglect; M = CTSPC - Sexual abuse; N = RFQ - Childhood family risk; O = ERGS - Hedonic goals; P = ERGS - Instrumental goals; Q = E-ERQ – Reappraisal; R = E-ERQ – Suppression; S = E-ERQ – Distraction; T = E-ERQ - Selective attention; U = E-ERQ - Situation selection.

**Figure S6.** Scatterplot using Obsessive-Compulsive PD as the dependent variable

**Figure S6.** Scatterplot using Obsessive-Compulsive PD as the dependent variable *(continued)*

**Figure S6.** Scatterplot using Obsessive-Compulsive PD as the dependent variable *(continued)*

**Figure S6.** Scatterplot using Obsessive-Compulsive PD as the dependent variable *(continued)*

*Note*. Scatterplots showing the distribution of the correlations between Obsessive-Compulsive PD and: A = LPFS - Individual personality dysfunction; B = LPFS - Interpersonal personality dysfunction; C = LPFS - Overall personality dysfunction; D = Socio-economic status during childhood; E = CTQ - Childhood maltreatment; F = QUIC - Childhood unpredictability; G = MSPSS - Childhood perceived social support; H = CTSPC - Non-violent discipline; I = CTSPC - Psychological aggression; J = CTSPC - Physical assault; K = CTSPC – Neglect; L = CTSPC - Sexual abuse; M = RFQ - Childhood family risk; N = ERGS - Hedonic goals; O = ERGS - Instrumental goals; P = E-ERQ – Reappraisal; Q = E-ERQ – Suppression; R = E-ERQ – Distraction; S = E-ERQ - Selective attention; T = E-ERQ - Situation selection.
