# Supplementary material for: The association between habitual emotion regulation, childhood adversity, and cluster C personality disorders symptomatology
Source: Front Psychol. 2026 Sep 3;17:1893753. doi: 10.3389/fpsyg.2026.1893753 (PMC13588754; doi:10.3389/fpsyg.2026.1893753)
Supplement: Supplementary file 4 [file Data_Sheet_4.docx]

**Supplementary material 4**

**1.3. Linearity and Homogeneity of variances**

**Figure S7.** Scatterplot using LPFS – Individual personality dysfunction as the dependent variable

**Figure S7.** Scatterplot using LPFS – Individual personality dysfunction as the dependent variable *(continued)*

**Figure S7.** Scatterplot using LPFS – Individual personality dysfunction as the dependent variable *(continued)*

**Figure S7.** Scatterplot using LPFS – Individual personality dysfunction as the dependent variable *(continued)*

*Note*. Scatterplots showing the distribution of the correlations between LPFS – Individual personality dysfunction and: A = LPFS - Interpersonal personality dysfunction; B = LPFS - Overall personality dysfunction; C = Socio-economic status during childhood; D = CTQ - Childhood maltreatment; E = QUIC - Childhood unpredictability; F = MSPSS - Childhood perceived social support; G = CTSPC - Non-violent discipline; H = CTSPC - Psychological aggression; I = CTSPC - Physical assault; J = CTSPC – Neglect; K = CTSPC - Sexual abuse; L = RFQ - Childhood family risk; M = ERGS - Hedonic goals; N = ERGS - Instrumental goals; O = E-ERQ – Reappraisal; P = E-ERQ – Suppression; Q = E-ERQ – Distraction; R = E-ERQ - Selective attention; S = E-ERQ - Situation selection.

**Figure S8.** Scatterplot using LPFS – Interpersonal personality dysfunction as the dependent variable

**Figure S8.** Scatterplot using LPFS – Interpersonal personality dysfunction as the dependent variable *(continued)*

**Figure S8.** Scatterplot using LPFS – Interpersonal personality dysfunction as the dependent variable *(continued)*

*Note*. Scatterplots showing the distribution of the correlations between LPFS – Interpersonal personality dysfunction and: A = LPFS - Overall personality dysfunction; B = Socio-economic status during childhood; C = CTQ - Childhood maltreatment; D = QUIC - Childhood unpredictability; E = MSPSS - Childhood perceived social support; F = CTSPC - Non-violent discipline; G = CTSPC - Psychological aggression; H = CTSPC - Physical assault; I = CTSPC – Neglect; J = CTSPC - Sexual abuse; K = RFQ - Childhood family risk; L = ERGS - Hedonic goals; M = ERGS - Instrumental goals; N = E-ERQ – Reappraisal; O = E-ERQ – Suppression; P = E-ERQ – Distraction; Q = E-ERQ - Selective attention; R = E-ERQ - Situation selection.

**Figure S9.** Scatterplot using LPFS – Overall personality dysfunction as the dependent variable

**Figure S9.** Scatterplot using LPFS – Overall personality dysfunction as the dependent variable *(continued)*

**Figure S9.** Scatterplot using LPFS – Overall personality dysfunction as the dependent variable *(continued)*

*Note*. Scatterplots showing the distribution of the correlations between LPFS – Overall personality dysfunction and: A = Socio-economic status during childhood; B = CTQ - Childhood maltreatment; C = QUIC - Childhood unpredictability; D = MSPSS - Childhood perceived social support; E = CTSPC - Non-violent discipline; F = CTSPC - Psychological aggression; G = CTSPC - Physical assault; H = CTSPC – Neglect; I = CTSPC - Sexual abuse; J = RFQ - Childhood family risk; K = ERGS - Hedonic goals; L = ERGS - Instrumental goals; M = E-ERQ – Reappraisal; N = E-ERQ – Suppression; O = E-ERQ – Distraction; P = E-ERQ - Selective attention; Q = E-ERQ - Situation selection.
