# Supplementary material for: The association between habitual emotion regulation, childhood adversity, and cluster C personality disorders symptomatology
Source: Front Psychol. 2026 Sep 3;17:1893753. doi: 10.3389/fpsyg.2026.1893753 (PMC13588754; doi:10.3389/fpsyg.2026.1893753)
Supplement: Supplementary file 5 [file Data_Sheet_5.docx]

**Supplementary material 5**

1.3. **Linearity and Homogeneity of variances**

**Figure S10.** Scatterplot using Socio-economic status during childhood as the dependent variable

**Figure S10.** Scatterplot using Socio-economic status during childhood as the dependent variable *(continued)*

**Figure S10.** Scatterplot using Socio-economic status during childhood as the dependent variable *(continued)*

*Note*. Scatterplots showing the distribution of the correlations between Socio-economic status during childhood and: A = CTQ - Childhood maltreatment; B = QUIC - Childhood unpredictability; C = MSPSS - Childhood perceived social support; D = CTSPC - Non-violent discipline; E = CTSPC - Psychological aggression; F = CTSPC - Physical assault; G = CTSPC – Neglect; H = CTSPC - Sexual abuse; I = RFQ - Childhood family risk; J = ERGS - Hedonic goals; K = ERGS - Instrumental goals; L = E-ERQ – Reappraisal; M = E-ERQ – Suppression; N = E-ERQ – Distraction; O = E-ERQ - Selective attention; P = E-ERQ - Situation selection.

**Figure S11.** Scatterplot using CTQ – Childhood maltreatment as the dependent variable

**Figure S11.** Scatterplot using CTQ – Childhood maltreatment as the dependent variable *(continued)*

**Figure S11.** Scatterplot using CTQ – Childhood maltreatment as the dependent variable *(continued)*

*Note*. Scatterplots showing the distribution of the correlations between CTQ – Childhood maltreatment and: A = QUIC - Childhood unpredictability; B = MSPSS - Childhood perceived social support; C = CTSPC - Non-violent discipline; D = CTSPC - Psychological aggression; E = CTSPC - Physical assault; F = CTSPC – Neglect; G = CTSPC - Sexual abuse; H = RFQ - Childhood family risk; I = ERGS - Hedonic goals; J = ERGS - Instrumental goals; K = E-ERQ – Reappraisal; L = E-ERQ – Suppression; M = E-ERQ – Distraction; N = E-ERQ - Selective attention; O = E-ERQ - Situation selection.

**Figure S12.** Scatterplot using QUIC - Childhood unpredictability as the dependent variable

**Figure S12.** Scatterplot using QUIC - Childhood unpredictability as the dependent variable *(continued)*

**Figure S12.** Scatterplot using QUIC - Childhood unpredictability as the dependent variable *(continued)*

*Note*. Scatterplots showing the distribution of the correlations between QUIC - Childhood unpredictability and: A = MSPSS - Childhood perceived social support; B = CTSPC - Non-violent discipline; C = CTSPC - Psychological aggression; D = CTSPC - Physical assault; E = CTSPC – Neglect; F = CTSPC - Sexual abuse; G = RFQ - Childhood family risk; H = ERGS - Hedonic goals; I = ERGS - Instrumental goals; J = E-ERQ – Reappraisal; K = E-ERQ – Suppression; L = E-ERQ – Distraction; M = E-ERQ - Selective attention; N = E-ERQ - Situation selection.

**Figure S13.** Scatterplot using MSPSS - Childhood perceived social support as the dependent variable

**Figure S13.** Scatterplot using MSPSS - Childhood perceived social support as the dependent variable *(continued)*

**Figure S13.** Scatterplot using MSPSS - Childhood perceived social support as the dependent variable *(continued)*

*Note*. Scatterplots showing the distribution of the correlations between MSPSS - Childhood perceived social support and: A = CTSPC - Non-violent discipline; B = CTSPC - Psychological aggression; C = CTSPC - Physical assault; D = CTSPC – Neglect; E = CTSPC - Sexual abuse; F = RFQ - Childhood family risk; G = ERGS - Hedonic goals; H = ERGS - Instrumental goals; I = E-ERQ – Reappraisal; J = E-ERQ – Suppression; K = E-ERQ – Distraction; L = E-ERQ - Selective attention; M = E-ERQ - Situation selection.

**Figure S14.** Scatterplot using CTSPC - Non-violent discipline as the dependent variable

**Figure S14.** Scatterplot using CTSPC - Non-violent discipline as the dependent variable *(continued)*

*Note*. Scatterplots showing the distribution of the correlations between CTSPC - Non-violent discipline and: A = CTSPC - Psychological aggression; B = CTSPC - Physical assault; C = CTSPC – Neglect; D = CTSPC - Sexual abuse; E = RFQ - Childhood family risk; F = ERGS - Hedonic goals; G = ERGS - Instrumental goals; H = E-ERQ – Reappraisal; I = E-ERQ – Suppression; J = E-ERQ – Distraction; K = E-ERQ - Selective attention; L = E-ERQ - Situation selection.

**Figure S15.** Scatterplot using CTSPC - Psychological aggression as the dependent variable

**Figure S15.** Scatterplot using CTSPC - Psychological aggression as the dependent variable *(continued)*

*Note*. Scatterplots showing the distribution of the correlations between CTSPC - Psychological aggression and: A = CTSPC - Physical assault; B = CTSPC – Neglect; C = CTSPC - Sexual abuse; D = RFQ - Childhood family risk; E = ERGS - Hedonic goals; F = ERGS - Instrumental goals; G = E-ERQ – Reappraisal; H = E-ERQ – Suppression; I = E-ERQ – Distraction; J = E-ERQ - Selective attention; K = E-ERQ - Situation selection.

**Figure S16.** using CTSPC – Physical assault as the dependent variable

**Figure S16.** Scatterplot using CTSPC – Physical assault as the dependent variable *(continued)*

*Note*. Scatterplots showing the distribution of the correlations between CTSPC - Physical assault and: A = CTSPC – Neglect; B = CTSPC - Sexual abuse; C = RFQ - Childhood family risk; D = ERGS - Hedonic goals; E = ERGS - Instrumental goals; F = E-ERQ – Reappraisal; G = E-ERQ – Suppression; H = E-ERQ – Distraction; I = E-ERQ - Selective attention; J = E-ERQ - Situation selection.

**Figure S17.** Scatterplot using CTSPC – Neglect as the dependent variable

**Figure S17.** Scatterplot using CTSPC – Neglect as the dependent variable *(continued)*

*Note*. Scatterplots showing the distribution of the correlations between CTSPC - Neglect and: A = CTSPC - Sexual abuse; B = RFQ - Childhood family risk; C = ERGS - Hedonic goals; D = ERGS - Instrumental goals; E = E-ERQ – Reappraisal; F = E-ERQ – Suppression; G = E-ERQ – Distraction; H = E-ERQ - Selective attention; I = E-ERQ - Situation selection.

**Figure S18.** Scatterplot using CTSPC – Sexual abuse as the dependent variable

**Figure S18.** Scatterplot using CTSPC – Sexual abuse as the dependent variable *(continued)*

*Note*. Scatterplots showing the distribution of the correlations between CTSPC – Sexual abuse and: A = RFQ - Childhood family risk; B = ERGS - Hedonic goals; C = ERGS - Instrumental goals; D = E-ERQ – Reappraisal; E = E-ERQ – Suppression; F = E-ERQ – Distraction; G = E-ERQ - Selective attention; H = E-ERQ - Situation selection.

**Figure S19.** Scatterplot using RFQ - Childhood family risk as the dependent variable

**Figure S19.** Scatterplot using RFQ - Childhood family risk as the dependent variable *(continued)*

*Note*. Scatterplots showing the distribution of the correlations between RFQ - Childhood family risk and: A = ERGS - Hedonic goals; B = ERGS - Instrumental goals; C = E-ERQ – Reappraisal; D = E-ERQ – Suppression; E = E-ERQ – Distraction; F = E-ERQ - Selective attention; G = E-ERQ - Situation selection.

**Figure S21.** Scatterplot using ERGS - Hedonic goals as the dependent variable

*Note*. Scatterplots showing the distribution of the correlations between ERGS - Hedonic goals and: A = ERGS - Instrumental goals; B = E-ERQ – Reappraisal; C = E-ERQ – Suppression; D = E-ERQ – Distraction; E = E-ERQ - Selective attention; F = E-ERQ - Situation selection.

**Figure S22.** Scatterplot using ERGS - Instrumental goals as the dependent variable

*Note*. Scatterplots showing the distribution of the correlations between ERGS - Instrumental goals and: A = E-ERQ – Reappraisal; B = E-ERQ – Suppression; C = E-ERQ – Distraction; D = E-ERQ - Selective attention; E = E-ERQ - Situation selection.

**Figure S23.** Scatterplot using E-ERQ – Reappraisal as the dependent variable

*Note*. Scatterplots showing the distribution of the correlations between E-ERQ – Reappraisal and: A = E-ERQ – Suppression; B = E-ERQ – Distraction; C = E-ERQ - Selective attention; D = E-ERQ - Situation selection.

**Figure S24.** Scatterplot using E-ERQ – Suppression as the dependent variable

*Note*. Scatterplots showing the distribution of the correlations between E-ERQ – Suppression and: A = E-ERQ – Distraction; B = E-ERQ - Selective attention; C = E-ERQ - Situation selection.

**Figure S25.** Scatterplot using E-ERQ – Distraction as the dependent variable

*Note*. Scatterplots showing the distribution of the correlations between E-ERQ – Distraction and: A = E-ERQ - Selective attention; B = E-ERQ - Situation selection.

**Figure S26.** Scatterplot using E-ERQ – Selective attention as the dependent variable

*Note.* Scatterplots showing the distribution of the correlations between E-ERQ – Selective attention and E-ERQ - Situation selection.
