# Supplementary material for: The association between habitual emotion regulation, childhood adversity, and cluster C personality disorders symptomatology
Source: Front Psychol. 2026 Sep 3;17:1893753. doi: 10.3389/fpsyg.2026.1893753 (PMC13588754; doi:10.3389/fpsyg.2026.1893753)
Supplement: Supplementary file 6 [file Data_Sheet_6.docx]

**Supplementary material 6**

**2. Spearman rank-order correlations**

**Table S2.** Spearman correlation (N = 141)

|  | AVPD | DPD | OCPD | LPFS Individ | LPFS Interpers | LPFS Total | SES child | CTQ Total | QUIC Total | MSPSS Total | CTSPC NVD | CTSPC Psych aggress | CTSPC Physical assault | CTSPC Neglect | CTSPC Sexual abuse | RFQ Total | ERGS HG | ERGS IG | E-ERQ R | E-ERQ S | E-ERQ D | E-ERQ SA | E-ERQ SS |
| --- | --- | --- | --- | --- | --- | --- | --- | --- | --- | --- | --- | --- | --- | --- | --- | --- | --- | --- | --- | --- | --- | --- | --- |
| AVPD | 1 |  |  |  |  |  |  |  |  |  |  |  |  |  |  |  |  |  |  |  |  |  |  |
| DPD | .457** | 1 |  |  |  |  |  |  |  |  |  |  |  |  |  |  |  |  |  |  |  |  |  |
| OCPD | -.282** | -.265* | 1 |  |  |  |  |  |  |  |  |  |  |  |  |  |  |  |  |  |  |  |  |
| LPFS - Individ | .449** | .532** | -.053 | 1 |  |  |  |  |  |  |  |  |  |  |  |  |  |  |  |  |  |  |  |
| LPFS - Interpers | .391** | .306** | .049 | .474** | 1 |  |  |  |  |  |  |  |  |  |  |  |  |  |  |  |  |  |  |
| LPFS - Total | .506** | .502** | -.007 | .883** | .813** | 1 |  |  |  |  |  |  |  |  |  |  |  |  |  |  |  |  |  |
| SES child | .124 | -.014 | .080 | .108 | .000 | .058 | 1 |  |  |  |  |  |  |  |  |  |  |  |  |  |  |  |  |
| CTQ - Total | .019 | .072 | -.037 | .125 | .286** | .225* | .011 | 1 |  |  |  |  |  |  |  |  |  |  |  |  |  |  |  |
| QUIC - Total | .115 | .159 | .034 | .252* | .237* | .280** | .056 | .718** | 1 |  |  |  |  |  |  |  |  |  |  |  |  |  |  |
| MSPSS - Total | -.154 | -.113 | .076 | -.254* | -.360** | -.360** | -.024 | -.708** | -.566** | 1 |  |  |  |  |  |  |  |  |  |  |  |  |  |
| CTSPC - NVD | -.067 | -.055 | .109 | -.100 | .059 | -.037 | .080 | .173* | .004 | -.080 | 1 |  |  |  |  |  |  |  |  |  |  |  |  |
| CTSPC - Psych aggress | .021 | -.033 | .054 | .133 | .193* | .190* | -.021 | .791** | .597** | -.618** | .279** | 1 |  |  |  |  |  |  |  |  |  |  |  |
| CTSPC - Physical assault | -.084 | -.069 | .073 | .023 | .227* | .125 | .023 | .696** | .399** | -.485** | .476** | .753** | 1 |  |  |  |  |  |  |  |  |  |  |
| CTSPC - Neglect | .078 | .146 | -.065 | .164 | .168* | .179* | .037 | .670** | .634** | -.595** | .067 | .565** | .428** | 1 |  |  |  |  |  |  |  |  |  |
| CTSPC - Sexual abuse | .020 | .028 | -.046 | .054 | .101 | .089 | .132 | .290** | .285** | -.091 | .058 | .246* | .209* | .190* | 1 |  |  |  |  |  |  |  |  |
| RFQ - Total | .059 | .156 | -.081 | .189* | .194* | .222* | .077 | .829** | .759** | -.658** | .071 | .773** | .565** | .691** | .203* | 1 |  |  |  |  |  |  |  |
| ERGS - HG | .060 | .099 | .039 | .228* | .161 | .232* | -.055 | .010 | .055 | .035 | .174* | -.051 | .066 | .015 | .038 | -.084 | 1 |  |  |  |  |  |  |
| ERGS - IG | .270** | .311** | -.036 | .248* | .071 | .199* | .034 | .052 | .063 | .021 | .103 | .052 | .038 | .064 | .125 | .080 | .223* | 1 |  |  |  |  |  |
| E-ERQ - R | -.257* | -.218* | .161 | -.268** | -.071 | -.215* | -.127 | -.201* | -.142 | .181* | .044 | -.219* | -.112 | -.116 | .114 | -.213* | .075 | .126 | 1 |  |  |  |  |
| E-ERQ - S | .299** | .145 | .016 | .254* | .309** | .314** | -.026 | .127 | .133 | -.194* | .057 | .112 | .064 | .094 | .060 | .096 | .164 | .266** | .159 | 1 |  |  |  |
| E-ERQ - D | .116 | .095 | .055 | .098 | .196* | .167* | -.081 | .002 | .060 | -.001 | .073 | .028 | -.006 | -.020 | .163 | -.016 | .116 | .334** | .489** | .342** | 1 |  |  |
| E-ERQ - SA | .206* | .137 | .075 | .185* | .351** | .296** | -.003 | -.044 | .029 | -.018 | .145 | -.023 | -.002 | -.023 | .098 | -.065 | .191* | .228* | .257* | .382** | .472** | 1 |  |
| E-ERQ - SS | -.147 | -.086 | .234* | .000 | .066 | .037 | -.033 | -.100 | -.024 | .050 | .096 | -.053 | -.053 | -.052 | .043 | -.112 | .232* | .230* | .558** | .117 | .469** | .276** | 1 |

**p ≤ .05; **p ≤ .001; AVPD = Avoidant personality disorder; DPD = Dependent personality disorder; OCPD = Obsessive-compulsive personality disorder; LPFS - Individ = Individual personality dysfunction; LPFS - Interpers = Interpersonal personality dysfunction; LPFS - Total = Overall personality dysfunction; SES child = Socio-economic status during childhood; CTQ - Total = Childhood maltreatment; QUIC - Total = Childhood unpredictability; MSPSS - Total = Childhood perceived social support; CTSPC NVD = Non-violent discipline; CTSPC - Psych aggress = Psychological aggression; RFQ - Total = Childhood family risk; ERGS - HG = Hedonic goals; ERGS - IG = Instrumental goals; E-ERQ - R = Reappraisal; E-ERQ - S = Suppression; E-ERQ - D = Distraction; E-ERQ - SA - Selective attention; E-ERQ - SS = Situation selection.*

3. **Partial correlation analysis, controlling for age and sex**

**Table S3.** Partial correlations controlling for age and sex (N = 141)

|  | AVPD | DPD | OCPD | LPFS Individ | LPFS Interpers | LPFS Total | SES child | CTQ Total | QUIC Total | MSPSS Total | CTSPC NVD | CTSPC Psych aggress | CTSPC Physical assault | CTSPC Neglect | CTSPC Sexual abuse | RFQ Total | ERGS HG | ERGS IG | E-ERQ R | E-ERQ S | E-ERQ D | E-ERQ SA | E-ERQ SS | Age | Sex |
| --- | --- | --- | --- | --- | --- | --- | --- | --- | --- | --- | --- | --- | --- | --- | --- | --- | --- | --- | --- | --- | --- | --- | --- | --- | --- |
| AVPD | 1 |  |  |  |  |  |  |  |  |  |  |  |  |  |  |  |  |  |  |  |  |  |  |  |  |
| DPD | .539** | 1 |  |  |  |  |  |  |  |  |  |  |  |  |  |  |  |  |  |  |  |  |  |  |  |
| OCPD | -.297** | -.285** | 1 |  |  |  |  |  |  |  |  |  |  |  |  |  |  |  |  |  |  |  |  |  |  |
| LPFS - Individ | .499** | .592** | -.057 | 1 |  |  |  |  |  |  |  |  |  |  |  |  |  |  |  |  |  |  |  |  |  |
| LPFS - Interpers | .446** | .301** | .063 | .529** | 1 |  |  |  |  |  |  |  |  |  |  |  |  |  |  |  |  |  |  |  |  |
| LPFS - Total | .542** | .526** | -.003 | .899** | .847** | 1 |  |  |  |  |  |  |  |  |  |  |  |  |  |  |  |  |  |  |  |
| SES child | .097 | -.011 | .114 | .077 | -.003 | .047 | 1 |  |  |  |  |  |  |  |  |  |  |  |  |  |  |  |  |  |  |
| CTQ - Total | .105 | .070 | -.058 | .148 | .307** | .251* | .019 | 1 |  |  |  |  |  |  |  |  |  |  |  |  |  |  |  |  |  |
| QUIC - Total | .193* | .161 | -.001 | .206* | .246* | .256* | .066 | .716** | 1 |  |  |  |  |  |  |  |  |  |  |  |  |  |  |  |  |
| MSPSS - Total | -.224* | -.112 | .093 | -.252* | -.363** | -.345** | -.029 | -.701** | -.582** | 1 |  |  |  |  |  |  |  |  |  |  |  |  |  |  |  |
| CTSPC - NVD | -.048 | -.085 | .149 | -.072 | .055 | -.017 | .098 | .152 | .044 | -.042 | 1 |  |  |  |  |  |  |  |  |  |  |  |  |  |  |
| CTSPC - Psych aggress | .104 | -.033 | .064 | .135 | .210* | .193* | -.020 | .769** | .595** | -.610** | .281** | 1 |  |  |  |  |  |  |  |  |  |  |  |  |  |
| CTSPC - Physical assault | .017 | -.054 | .078 | .071 | .250* | .173* | .018 | .686** | .422** | -.468** | .454** | .731** | 1 |  |  |  |  |  |  |  |  |  |  |  |  |
| CTSPC - Neglect | .107 | .141 | -.085 | .125 | .175* | .168* | .033 | .677** | .634** | -.589** | .068 | .554** | .443** | 1 |  |  |  |  |  |  |  |  |  |  |  |
| CTSPC - Sexual abuse | .095 | .049 | -.029 | .090 | .148 | .133 | .136 | .417** | .304** | -.205* | .089 | .276** | .281** | .265* | 1 |  |  |  |  |  |  |  |  |  |  |
| RFQ - Total | .133 | .150 | -.071 | .178* | .204* | .217* | .081 | .807** | .762** | -.659** | .057 | .769** | .561** | .698** | .253* | 1 |  |  |  |  |  |  |  |  |  |
| ERGS - HG | .036 | .055 | .031 | .210* | .182* | .225* | -.042 | .002 | .000 | .038 | .205* | -.052 | .085 | .018 | .104 | -.106 | 1 |  |  |  |  |  |  |  |  |
| ERGS - IG | .326** | .354** | -.086 | .251* | .100 | .209* | .020 | .080 | .058 | .001 | .136 | .076 | .101 | .092 | .108 | .077 | .208* | 1 |  |  |  |  |  |  |  |
| E-ERQ - R | -.229* | -.220* | .110 | -.288** | -.080 | -.222* | -.099 | -.198* | -.139 | .209* | .057 | -.240* | -.125 | -.130 | .068 | -.224* | .131 | .114 | 1 |  |  |  |  |  |  |
| E-ERQ - S | .347** | .140 | .010 | .263* | .335** | .338** | -.045 | .135 | .181* | -.221* | .092 | .130 | .126 | .099 | .066 | .101 | .169* | .276** | .147 | 1 |  |  |  |  |  |
| E-ERQ - D | .131 | .124 | -.003 | .102 | .197* | .165 | -.086 | .031 | .085 | -.031 | .077 | .043 | .004 | -.031 | .095 | -.003 | .146 | .315** | .527** | .384** | 1 |  |  |  |  |
| E-ERQ - SA | .201* | .164 | .057 | .197* | .320** | .289** | -.001 | -.054 | -.008 | .012 | .201* | -.043 | -.007 | -.051 | .096 | -.099 | .211* | .266* | .274** | .389** | .479** | 1 |  |  |  |
| E-ERQ - SS | -.159 | -.089 | .198* | -.054 | .063 | -.001 | -.025 | -.113 | -.047 | .094 | .152 | -.064 | -.065 | -.097 | -.020 | -.138 | .288** | .231* | .541** | .116 | .473** | .313** | 1 |  |  |
| Age | -.152 | .069 | -.015 | -.249* | .099 | -.109 | -.037 | -.058 | -.175* | .011 | .222* | -.055 | .044 | -.102 | -.068 | -.070 | .008 | -.073 | -.015 | .030 | -.023 | -.035 | -.132 | 1 |  |
| Sex | -.108 | .080 | .035 | -.048 | .129 | .036 | -.092 | .124 | .055 | -.145 | .098 | .076 | .172* | .142 | -.087 | .063 | .101 | -.094 | .003 | .015 | -.136 | -.002 | .030 | .111 | 1 |

**p ≤ .05; **p ≤ .001; AVPD = Avoidant personality disorder; DPD = Dependent personality disorder; OCPD = Obsessive-compulsive personality disorder; LPFS - Individ = Individual personality dysfunction; LPFS - Interpers = Interpersonal personality dysfunction; LPFS - Total = Overall personality dysfunction; SES child = Socio-economic status during childhood; CTQ - Total = Childhood maltreatment; QUIC - Total = Childhood unpredictability; MSPSS - Total = Childhood perceived social support; CTSPC NVD = Non-violent discipline; CTSPC - Psych aggress = Psychological aggression; RFQ - Total = Childhood family risk; ERGS - HG = Hedonic goals; ERGS - IG = Instrumental goals; E-ERQ - R = Reappraisal; E-ERQ - S = Suppression; E-ERQ - D = Distraction; E-ERQ - SA - Selective attention; E-ERQ - SS = Situation selection.*

4. **Assumption checks for moderation**
   4.1. **Normality** **of residuals –** qq plots, histograms

**Figure S27.** Histograms of Avoidant PD residuals

*Note*. Histograms of Avoidant PD residuals diagnostics. A = ERGS – Hedonic goals; B = ERGS – Instrumental goals; C = E-ERQ – Reappraisal; D = E-ERQ – Suppression; E = E-ERQ – Distraction; F = E-ERQ – Selective attention; G = E-ERQ – Situation selection.

**Figure S28.** Normal Q-Q Plots of Avoidant PD residuals

**Figure S28.** Normal Q-Q Plots of Avoidant PD residuals *(continued)*

*Note*. Normal Q-Q plots of Avoidant PD residuals diagnostics. A = ERGS – Hedonic goals; B = ERGS – Instrumental goals; C = E-ERQ – Reappraisal; D = E-ERQ – Suppression; E = E-ERQ – Distraction; F = E-ERQ – Selective attention; G = E-ERQ – Situation selection.

**Figure S29.** Histograms of Dependent PD residuals

*Note*. Histograms of Dependent PD residuals diagnostics. A = ERGS – Hedonic goals; B = ERGS – Instrumental goals; C = E-ERQ – Reappraisal; D = E-ERQ – Suppression; E = E-ERQ – Distraction; F = E-ERQ – Selective attention; G = E-ERQ – Situation selection.

**Figure S30.** Normal Q-Q Plots of Dependent PD residuals

**Figure S30.** Normal Q-Q Plots of Dependent PD residuals *(continued)*

*Note*. Normal Q-Q plots of Dependent PD residuals diagnostics. A = ERGS – Hedonic goals; B = ERGS – Instrumental goals; C = E-ERQ – Reappraisal; D = E-ERQ – Suppression; E = E-ERQ – Distraction; F = E-ERQ – Selective attention; G = E-ERQ – Situation selection.

**Figure S31.** Histograms of Obsessive-compulsive PD residuals

*Note*. Histograms of Obsessive-compulsive PD residuals diagnostics. A = ERGS – Hedonic goals; B = ERGS – Instrumental goals; C = E-ERQ – Reappraisal; D = E-ERQ – Suppression; E = E-ERQ – Distraction; F = E-ERQ – Selective attention; G = E-ERQ – Situation selection.

**Figure S32.** Normal Q-Q Plots of Obsessive-compulsive PD residuals

**Figure S32.** Normal Q-Q Plots of Obsessive-compulsive PD residuals *(continued)*

*Note*. Normal Q-Q plots of Obsessive-compulsive PD residuals diagnostics. A = ERGS – Hedonic goals; B = ERGS – Instrumental goals; C = E-ERQ – Reappraisal; D = E-ERQ – Suppression; E = E-ERQ – Distraction; F = E-ERQ – Selective attention; G = E-ERQ – Situation selection.

4.2. **Linearity and homoscedasticity**

**Figure S33.** Scatterplot of Avoidant PD residuals

*Note*. Scatterplots of Avoidant PD residuals diagnostics. A = ERGS – Hedonic goals; B = ERGS – Instrumental goals; C = E-ERQ – Reappraisal; D = E-ERQ – Suppression; E = E-ERQ – Distraction; F = E-ERQ – Selective attention; G = E-ERQ – Situation selection.

**Figure S34.** Scatterplot of Dependent PD residuals

*Note*. Scatterplots of Dependent PD residuals diagnostics. A = ERGS – Hedonic goals; B = ERGS – Instrumental goals; C = E-ERQ – Reappraisal; D = E-ERQ – Suppression; E = E-ERQ – Distraction; F = E-ERQ – Selective attention; G = E-ERQ – Situation selection.

**Figure S35.** Scatterplot of Obsessive-compulsive PD residuals

*Note*. Scatterplots of Obsessive-compulsive PD residuals diagnostics. A = ERGS – Hedonic goals; B = ERGS – Instrumental goals; C = E-ERQ – Reappraisal; D = E-ERQ – Suppression; E = E-ERQ – Distraction; F = E-ERQ – Selective attention; G = E-ERQ – Situation selection.

4.3. **Multicollinearity analysis**

**Table S4.** Collinearity statistics for avoidant personality disorder (N = 141)

| Factor | B | SE | t | *p* | Collinearity | |
| --- | --- | --- | --- | --- | --- | --- |
|  |  |  |  |  | Tolerance | VIF |
| Hedonic goals | -.203 | 1.537 | -0.132 | .895 | .102 | 9.777 |
| CTQ | .000 | 0.106 | -0.001 | .998 | .038 | 26.408 |
| Hedonic goals*CTQ | .006 | 0.026 | 0.231 | .818 | .028 | 35.627 |
| Instrumental goals | 2.299* | 1.079 | 2.131 | .035 | .114 | 8.770 |
| CTQ | .100 | 0.108 | 0.932 | .353 | .033 | 30.641 |
| Instrumental goals*CTQ | -.016 | 0.020 | -0.775 | .439 | .025 | 40.562 |
| Reappraisal | -.504 | 0.976 | -0.516 | .606 | .093 | 10.746 |
| CTQ | .037 | 0.085 | 0.434 | .665 | .057 | 17.639 |
| Reappraisal*CTQ | -.005 | 0.017 | -0.278 | .781 | .045 | 22.379 |
| Suppression | 1.381 | 0.793 | 1.741 | .084 | .117 | 8.513 |
| CTQ | .037 | 0.068 | 0.544 | .588 | .083 | 11.980 |
| Suppression*CTQ | -.005 | 0.015 | -0.362 | .718 | .046 | 21.871 |
| Distraction | .613 | 0.986 | 0.622 | .535 | .091 | 10.995 |
| CTQ | .034 | 0.089 | 0.380 | .704 | .053 | 18.720 |
| Distraction*CTQ | -.002 | 0.018 | -0.118 | .906 | .034 | 29.083 |
| Selective attention | 1.332 | 0.766 | 1.740 | .084 | .126 | 7.909 |
| CTQ | .073 | 0.055 | 1.325 | .187 | .135 | 7.403 |
| Selective attention*CTQ | -.012 | 0.014 | -0.904 | .368 | .073 | 13.628 |
| Situation selection | .641 | 0.834 | 0.768 | .444 | .101 | 9.909 |
| CTQ | .116 | 0.074 | 1.565 | .120 | .076 | 13.113 |
| Situation selection*CTQ | -.020 | 0.015 | -1.334 | .184 | .050 | 19.991 |

**p ≤.05; CTQ = Childhood maltreatment.*

**Table S5.** Collinearity statistics for dependent personality disorder (N = 141)

| Factor | B | SE | t | *p* | Collinearity | |
| --- | --- | --- | --- | --- | --- | --- |
|  |  |  |  |  | Tolerance | VIF |
| Hedonic goals | .087 | 1.770 | 0.049 | .961 | .102 | 9.777 |
| CTQ | -.003 | 0.122 | -0.021 | .983 | .038 | 26.408 |
| Hedonic goals*CTQ | .006 | 0.030 | 0.193 | .847 | .028 | 35.627 |
| Instrumental goals | 2.180 | 1.245 | 1.751 | .082 | .114 | 8.770 |
| CTQ | .058 | 0.124 | 0.468 | .640 | .033 | 30.641 |
| Instrumental goals*CTQ | -.008 | 0.023 | -0.361 | .719 | .025 | 40.562 |
| Reappraisal | -1.328 | 1.126 | -1.180 | .240 | .093 | 10.746 |
| CTQ | -.031 | 0.098 | -0.313 | .755 | .057 | 17.639 |
| Reappraisal*CTQ | .008 | 0.020 | 0.420 | .675 | .045 | 22.379 |
| Suppression | 1.062 | 0.959 | 1.108 | .270 | .117 | 8.513 |
| CTQ | .063 | 0.082 | 0.765 | .446 | .083 | 11.980 |
| Suppression*CTQ | -.011 | 0.018 | -0.597 | .551 | .046 | 21.871 |
| Distraction | .358 | 1.142 | 0.313 | .755 | .091 | 10.995 |
| CTQ | .012 | 0.103 | 0.119 | .905 | .053 | 18.720 |
| Distraction*CTQ | .002 | 0.021 | 0.081 | .935 | .034 | 29.083 |
| Selective attention | .632 | 0.893 | 0.707 | .481 | .126 | 7.909 |
| CTQ | .024 | 0.064 | 0.371 | .712 | .135 | 7.403 |
| Selective attention*CTQ | .000 | 0.016 | -0.009 | .993 | .073 | 13.628 |
| Situation selection | 1.313 | 0.962 | 1.364 | .175 | .101 | 9.909 |
| CTQ | .165 | 0.085 | 1.934 | .055 | .076 | 13.113 |
| Situation selection*CTQ | -.031 | 0.017 | -1.788 | .076 | .050 | 19.991 |

*CTQ = Childhood maltreatment.*

**Table S6.** Collinearity statistics for obsessive-compulsive personality disorder (N = 141)

| Factor | b | SE | t | *p* | Collinearity | |
| --- | --- | --- | --- | --- | --- | --- |
|  |  |  |  |  | Tolerance | VIF |
| Hedonic goals | 3.560* | 1.209 | 2.945 | .004 | .102 | 9.777 |
| CTQ | .233* | 0.084 | 2.782 | .006 | .038 | 26.408 |
| Hedonic goals*CTQ | -.061* | 0.021 | -2.963 | .004 | .028 | 35.627 |
| Instrumental goals | .131 | 0.926 | 0.142 | .887 | .114 | 8.770 |
| CTQ | .037 | 0.093 | 0.400 | .690 | .033 | 30.641 |
| Instrumental goals*CTQ | -.009 | 0.017 | -0.506 | .613 | .025 | 40.562 |
| Reappraisal | .866 | 0.805 | 1.076 | .284 | .093 | 10.746 |
| CTQ | .044 | 0.070 | 0.627 | .532 | .057 | 17.639 |
| Reappraisal*CTQ | -.010 | 0.014 | -0.739 | .461 | .045 | 22.379 |
| Suppression | .853 | 0.677 | 1.260 | .210 | .117 | 8.513 |
| CTQ | .059 | 0.058 | 1.029 | .305 | .083 | 11.980 |
| Suppression*CTQ | -.016 | 0.012 | -1.269 | .207 | .046 | 21.871 |
| Distraction | .840 | 0.804 | 1.044 | .298 | .091 | 10.995 |
| CTQ | .069 | 0.072 | 0.948 | .345 | .053 | 18.720 |
| Distraction*CTQ | -.016 | 0.014 | -1.120 | .265 | .034 | 29.083 |
| Selective attention | .849 | 0.633 | 1.341 | .182 | .126 | 7.909 |
| CTQ | .040 | 0.045 | 0.890 | .375 | .135 | 7.403 |
| Selective attention*CTQ | -.013 | 0.011 | -1.187 | .237 | .073 | 13.628 |
| Situation selection | .114 | 0.673 | 0.169 | .866 | .101 | 9.909 |
| CTQ | -.041 | 0.060 | -0.696 | .487 | .076 | 13.113 |
| Situation selection*CTQ | .007 | 0.012 | 0.611 | .542 | .050 | 19.991 |

**p ≤.05; CTQ = Childhood maltreatment.*

5. **Moderation analysis, controlling for age and sex with HC3 heteroscedasticity**
   5.1. **Moderation analysis using avoidant PD as the outcome**

**Table S7.** Moderation results for avoidant personality disorder (N = 141).

| Factor | b | SE  (HC3) | t | *p* | 95% CI | |
| --- | --- | --- | --- | --- | --- | --- |
|  |  |  |  |  | LL | UL |
| Hedonic goals | .173 | 0.495 | 0.350 | .726 | -0.806 | 1.153 |
| CTQ | .024 | 0.023 | 1.038 | .300 | -0.022 | 0.071 |
| Hedonic goals*CTQ | .013 | 0.027 | 0.480 | .631 | -0.041 | 0.067 |
| Age | -.067 | 0.048 | -1.376 | .171 | -0.163 | 0.029 |
| Sex | -1.587 | 1.555 | -1.020 | .309 | -4.663 | 1.489 |
| Instrumental goals | 1.411** | 0.399 | 3.533 | .0006 | 0.621 | 2.202 |
| CTQ | .020 | 0.024 | 0.870 | .385 | -0.026 | 0.068 |
| Instrumental goals*CTQ | -.012 | 0.023 | -0.537 | .591 | -0.059 | 0.034 |
| Age | -.056 | 0.045 | -1.241 | .216 | -0.146 | 0.033 |
| Sex | -.961 | 1.370 | -0.701 | .484 | -3.671 | 1.748 |
| Reappraisal | -.766* | 0.336 | -2.281 | .024 | -1.431 | -0.102 |
| CTQ | .014 | 0.023 | 0.632 | .528 | -0.031 | 0.061 |
| Reappraisal*CTQ | .0002 | 0.018 | 0.013 | .989 | -0.035 | 0.036 |
| Age | -.068 | 0.048 | -1.425 | .156 | -0.164 | 0.026 |
| Sex | -1.392 | 1.600 | -0.870 | .385 | -4.557 | 1.772 |
| Suppression | 1.126** | 0.283 | 3.969 | .0001 | 0.565 | 1.688 |
| CTQ | .014 | 0.021 | 0.653 | .514 | -0.029 | 0.057 |
| Suppression*CTQ | -.001 | 0.014 | -0.125 | .900 | -0.030 | 0.027 |
| Age | -.071 | 0.051 | -1.389 | .166 | -0.173 | 0.030 |
| Sex | -1.437 | 1.312 | -1.095 | .275 | -4.033 | 1.158 |
| Distraction | .448 | 0.332 | 1.348 | .179 | -0.209 | 1.106 |
| CTQ | .024 | 0.024 | 1.014 | .312 | -0.023 | 0.071 |
| Distraction*CTQ | .0006 | 0.021 | 0.028 | .977 | -0.041 | 0.042 |
| Age | -.065 | 0.047 | -1.371 | .172 | -0.159 | 0.028 |
| Sex | -1.234 | 1.461 | -0.845 | .399 | -4.124 | 1.655 |
| Selective attention | .669* | 0.276 | 2.423 | .016 | 0.123 | 1.216 |
| CTQ | .028 | 0.022 | 1.228 | .221 | -0.017 | 0.073 |
| Selective attention*CTQ | -.009 | 0.014 | -0.685 | .493 | -0.038 | 0.018 |
| Age | -.062 | 0.046 | -1.332 | .185 | -0.154 | 0.030 |
| Sex | -1.379 | 1.459 | -0.944 | .346 | -4.265 | 1.507 |
| Situation selection | -.469 | 0.281 | -1.670 | .097 | -1.025 | 0.086 |
| CTQ | .020 | 0.024 | 0.828 | .408 | -0.027 | 0.068 |
| Situation selection*CTQ | -.015 | 0.018 | -0.888 | .376 | -0.051 | 0.019 |
| Age | -.071 | 0.048 | -1.472 | .143 | -0.167 | 0.024 |
| Sex | -1.293 | 1.518 | -0.851 | .395 | -4.296 | 1.709 |

**p ≤.05; **p ≤ .001; CTQ = Childhood maltreatment.*

5.2. **Moderation analysis using dependent PD as the outcome**

**Table S8.** Moderation results for dependent personality disorder (N = 141).

| Factor | b | SE  (HC3) | t | *p* | 95% CI | |
| --- | --- | --- | --- | --- | --- | --- |
|  |  |  |  |  | LL | UL |
| Hedonic goals | .364 | 0.591 | 0.615 | .539 | -0.805 | 1.533 |
| CTQ | .019 | 0.024 | 0.787 | .432 | -0.029 | 0.068 |
| Hedonic goals*CTQ | .001 | 0.030 | 0.057 | .953 | -0.058 | 0.062 |
| Age | .037 | 0.054 | 0.676 | .500 | -0.071 | 0.145 |
| Sex | .938 | 1.400 | 0.670 | .503 | -1.830 | 3.707 |
| Instrumental goals | 1.815** | 0.421 | 4.309 | .0000 | 0.982 | 2.648 |
| CTQ | .013 | 0.023 | 0.587 | .558 | -0.031 | 0.058 |
| Instrumental goals*CTQ | -.012 | 0.022 | -0.554 | .580 | -0.057 | 0.032 |
| Age | .048 | 0.048 | 1.001 | .318 | -0.047 | 0.145 |
| Sex | 1.675 | 1.248 | 1.342 | .181 | -0.793 | 4.144 |
| Reappraisal | -.888* | 0.338 | -2.625 | .009 | -1.557 | -0.219 |
| CTQ | -.007 | 0.025 | 0.303 | .762 | -0.041 | 0.057 |
| Reappraisal*CTQ | .004 | 0.019 | 0.250 | .802 | -0.033 | 0.043 |
| Age | .033 | 0.053 | 0.621 | .535 | -0.072 | 0.138 |
| Sex | 1.088 | 1.449 | 0.751 | .453 | -1.778 | 3.955 |
| Suppression | .492 | 0.362 | 1.358 | .176 | -0.224 | 1.210 |
| CTQ | .014 | 0.024 | 0.607 | .544 | -0.033 | 0.062 |
| Suppression*CTQ | -.012 | 0.019 | -0.665 | .506 | -0.051 | 0.025 |
| Age | .035 | 0.057 | 0.619 | .536 | -0.077 | 0.148 |
| Sex | 1.164 | 1.405 | 0.828 | .408 | -1.614 | 3.943 |
| Distraction | .497 | 0.354 | 1.403 | .162 | -0.203 | 1.199 |
| CTQ | .018 | 0.024 | 0.771 | .441 | -0.029 | 0.066 |
| Distraction*CTQ | -.0001 | 0.021 | -0.002 | .998 | -0.042 | 0.042 |
| Age | .037 | 0.053 | 0.698 | .486 | -0.068 | 0.143 |
| Sex | 1.310 | 1.369 | 0.957 | .340 | -1.397 | 4.018 |
| Selective attention | .633 | 0.333 | 1.898 | .059 | -0.026 | 1.293 |
| CTQ | .022 | 0.024 | 0.970 | .366 | -0.026 | 0.070 |
| Selective attention*CTQ | -.002 | 0.017 | -0.114 | .909 | -0.036 | 0.032 |
| Age | .040 | 0.052 | 0.770 | .442 | -0.063 | 0.145 |
| Sex | 1.038 | 1.434 | 0.723 | .470 | -1.799 | 3.875 |
| Situation selection | -.300 | 0.331 | -0.909 | .364 | -0.955 | 0.353 |
| CTQ | .014 | 0.024 | 0.596 | .551 | -0.033 | 0.062 |
| Situation selection*CTQ | -.033 | 0.018 | -1.877 | .062 | -0.069 | 0.001 |
| Age | .040 | 0.053 | 0.757 | .450 | -0.065 | 0.146 |
| Sex | 1.250 | 1.364 | 0.916 | .360 | -1.447 | 3.949 |

**p ≤.05; **p ≤ .001; CTQ = Childhood maltreatment.*

5.3. **Moderation analysis using obsessive-compulsive PD as the outcome**

**Table S9.** Moderation results for obsessive-compulsive personality disorder (N = 141).

| Factor | b | SE  (HC3) | t | *p* | 95% CI | |
| --- | --- | --- | --- | --- | --- | --- |
|  |  |  |  |  | LL | UL |
| Hedonic goals | .288 | 0.416 | 0.693 | .489 | -0.534 | 1.111 |
| CTQ | -.007 | 0.016 | -0.443 | .657 | -0.040 | 0.025 |
| Hedonic goals*CTQ | -.062* | 0.024 | -2.580 | .010 | -0.111 | -0.014 |
| Age | -.001 | 0.035 | -0.054 | .956 | -0.072 | 0.068 |
| Sex | .733 | 0.991 | 0.739 | .460 | -1.227 | 2.694 |
| Instrumental goals | -.324 | 0.373 | -0.868 | .386 | -1.062 | 0.414 |
| CTQ | -.009 | 0.016 | -0.535 | .593 | -0.042 | 0.024 |
| Instrumental goals*CTQ | -.009 | 0.017 | -0.543 | .587 | -0.044 | 0.025 |
| Age | -.011 | 0.035 | -0.313 | .754 | -0.080 | 0.058 |
| Sex | .477 | 0.970 | 0.492 | .623 | -1.441 | 2.396 |
| Reappraisal | .315 | 0.250 | 1.258 | .210 | -0.180 | 0.811 |
| CTQ | -.007 | 0.017 | -0.405 | .686 | -0.042 | 0.028 |
| Reappraisal*CTQ | -.011 | 0.018 | -0.621 | .535 | -0.048 | 0.025 |
| Age | -.007 | 0.035 | -0.198 | .842 | -0.076 | 0.062 |
| Sex | .591 | 1.042 | 0.567 | .571 | -1.469 | 2.652 |
| Suppression | .019 | 0.238 | 0.081 | .935 | -0.451 | 0.490 |
| CTQ | -.011 | 0.017 | -0.662 | .508 | -0.047 | 0.023 |
| Suppression*CTQ | -.016 | 0.014 | -1.183 | .238 | -0.044 | 0.011 |
| Age | -.008 | 0.035 | -0.232 | .816 | -0.077 | 0.061 |
| Sex | .653 | 0.971 | 0.672 | .502 | -1.267 | 2.573 |
| Distraction | -.001 | 0.234 | -0.004 | .996 | -0.465 | 0.463 |
| CTQ | -.009 | 0.017 | -0.537 | .591 | -0.044 | 0.025 |
| Distraction*CTQ | -.016 | 0.015 | -1.053 | .294 | -0.046 | 0.014 |
| Age | -.006 | 0.035 | -0.178 | .858 | -0.076 | 0.063 |
| Sex | .513 | 1.015 | 0.505 | .613 | -1.494 | 2.521 |
| Selective attention | .142 | 0.238 | 0.596 | .551 | -0.328 | 0.612 |
| CTQ | -.010 | 0.018 | -0.603 | .547 | -0.046 | 0.024 |
| Selective attention*CTQ | -.014 | 0.015 | -0.937 | .350 | -0.045 | 0.016 |
| Age | -.009 | 0.034 | -0.260 | .794 | -0.078 | 0.060 |
| Sex | .687 | 0.989 | 0.694 | .488 | -1.269 | 2.644 |
| Situation selection | .501* | 0.207 | 2.415 | .017 | 0.091 | 0.912 |
| CTQ | -.006 | 0.015 | -0.423 | .672 | -0.038 | 0.024 |
| Situation selection*CTQ | .007 | 0.012 | 0.569 | .570 | -0.017 | 0.031 |
| Age | -.0003 | 0.034 | -0.007 | .993 | -0.068 | 0.068 |
| Sex | .341 | 0.959 | 0.355 | .722 | -1.557 | 2.239 |

**p ≤.05; CTQ = Childhood maltreatment.*

6. **Partial correlation, controlling for age in the case of female sample**

**Table S10.** Partial correlations controlling for age (N = 125)

|  | AVPD | DPD | OCPD | LPFS Individ | LPFS Interpers | LPFS Total | SES child | CTQ Total | QUIC Total | MSPSS Total | CTSPC NVD | CTSPC Psych aggress | CTSPC Physical assault | CTSPC Neglect | CTSPC Sexual abuse | RFQ Total | ERGS HG | ERGS IG | E-ERQ R | E-ERQ S | E-ERQ D | E-ERQ SA | E-ERQ SS | Age |
| --- | --- | --- | --- | --- | --- | --- | --- | --- | --- | --- | --- | --- | --- | --- | --- | --- | --- | --- | --- | --- | --- | --- | --- | --- |
| AVPD | 1 |  |  |  |  |  |  |  |  |  |  |  |  |  |  |  |  |  |  |  |  |  |  |  |
| DPD | .518** | 1 |  |  |  |  |  |  |  |  |  |  |  |  |  |  |  |  |  |  |  |  |  |  |
| OCPD | -.273* | -.249* | 1 |  |  |  |  |  |  |  |  |  |  |  |  |  |  |  |  |  |  |  |  |  |
| LPFS - Individ | .478** | .559** | -.003 | 1 |  |  |  |  |  |  |  |  |  |  |  |  |  |  |  |  |  |  |  |  |
| LPFS - Interpers | .436** | .269* | .130 | .509** | 1 |  |  |  |  |  |  |  |  |  |  |  |  |  |  |  |  |  |  |  |
| LPFS - Total | .528** | .492** | .066 | .894** | .840** | 1 |  |  |  |  |  |  |  |  |  |  |  |  |  |  |  |  |  |  |
| SES child | .075 | -.014 | .085 | .058 | -.030 | .021 | 1 |  |  |  |  |  |  |  |  |  |  |  |  |  |  |  |  |  |
| CTQ - Total | .044 | .011 | -.032 | .116 | .271* | .214* | .025 | 1 |  |  |  |  |  |  |  |  |  |  |  |  |  |  |  |  |
| QUIC - Total | .144 | .090 | .057 | .178* | .231* | .232* | .079 | .696** | 1 |  |  |  |  |  |  |  |  |  |  |  |  |  |  |  |
| MSPSS - Total | -.186* | -.060 | .040 | -.237* | -.334** | -.323** | -.057 | -.690** | -.563** | 1 |  |  |  |  |  |  |  |  |  |  |  |  |  |  |
| CTSPC - NVD | -.091 | -.112 | .187* | -.067 | .026 | -.029 | .149 | .112 | .011 | -.007 | 1 |  |  |  |  |  |  |  |  |  |  |  |  |  |
| CTSPC - Psych aggress | .052 | -.090 | .112 | .123 | .174 | .168 | .000 | .774** | .603** | -.600** | .224* | 1 |  |  |  |  |  |  |  |  |  |  |  |  |
| CTSPC - Physical assault | -.066 | -.136 | .171 | .042 | .220* | .141 | .035 | .691** | .389** | -.460** | .423** | .722** | 1 |  |  |  |  |  |  |  |  |  |  |  |
| CTSPC - Neglect | .105 | .141 | -.066 | .116 | .158 | .155 | .038 | .673** | .637** | -.603** | .046 | .553** | .415** | 1 |  |  |  |  |  |  |  |  |  |  |
| CTSPC - Sexual abuse | .095 | .020 | -.039 | .080 | .161 | .134 | .093 | .421** | .289** | -.226* | .086 | .265* | .259* | .241* | 1 |  |  |  |  |  |  |  |  |  |
| RFQ - Total | .076 | .101 | -.034 | .146 | .161 | .175 | .105 | .809** | .768** | -.657** | .013 | .771** | .553** | .689** | .220* | 1 |  |  |  |  |  |  |  |  |
| ERGS - HG | .007 | -.013 | .089 | .185* | .174 | .207* | -.008 | -.029 | -.042 | .063 | .183* | -.103 | .038 | .021 | .079 | -.135 | 1 |  |  |  |  |  |  |  |
| ERGS - IG | .285** | .318** | -.069 | .213* | .025 | .147 | .039 | .013 | -.014 | .067 | .093 | -.011 | .037 | .075 | .096 | -.018 | .202* | 1 |  |  |  |  |  |  |
| E-ERQ - R | -.289** | -.231* | .102 | -.307** | -.116 | -.253* | -.136 | -.253* | -.167 | .246* | .031 | -.315** | -.169 | -.161 | .023 | -.291** | .148 | .090 | 1 |  |  |  |  |  |
| E-ERQ - S | .285** | .079 | .086 | .245* | .316** | .319** | -.023 | .094 | .133 | -.172 | .055 | .079 | .054 | .097 | .084 | .044 | .161 | .231* | .143 | 1 |  |  |  |  |
| E-ERQ - D | .068 | .079 | .015 | .071 | .161 | .128 | -.104 | .003 | .054 | .008 | .049 | .001 | -.019 | -.003 | .102 | -.032 | .124 | .261* | .531** | .350** | 1 |  |  |  |
| E-ERQ - SA | .159 | .156 | .095 | .211* | .291** | .284** | -.016 | -.115 | -.060 | .058 | .172 | -.093 | -.062 | -.072 | .095 | -.134 | .197* | .260* | .254* | .370** | .460** | 1 |  |  |
| E-ERQ - SS | -.156 | -.076 | .184* | -.045 | .069 | .007 | -.047 | -.126 | -.032 | .095 | .157 | -.085 | -.060 | -.095 | -.049 | -.145 | .315** | .252* | .531** | .144 | .490** | .330** | 1 |  |
| Age | -.129 | .090 | -.068 | -.233* | .064 | -.117 | -.081 | -.067 | -.183* | .028 | .233* | -.042 | .070 | -.117 | -.0050- -.050 | -.085 | .065 .065 | -.086 | -.039 | .040 | -.036 | -.044 | -.139 | 1 |

**p ≤ .05; **p ≤ .001; AVPD = Avoidant personality disorder; DPD = Dependent personality disorder; OCPD = Obsessive-compulsive personality disorder; LPFS - Individ = Individual personality dysfunction; LPFS - Interpers = Interpersonal personality dysfunction; LPFS - Total = Overall personality dysfunction; SES child = Socio-economic status during childhood; CTQ - Total = Childhood maltreatment; QUIC - Total = Childhood unpredictability; MSPSS - Total = Childhood perceived social support; CTSPC NVD = Non-violent discipline; CTSPC - Psych aggress = Psychological aggression; RFQ - Total = Childhood family risk; ERGS - HG = Hedonic goals; ERGS - IG = Instrumental goals; E-ERQ - R = Reappraisal; E-ERQ - S = Suppression; E-ERQ - D = Distraction; E-ERQ - SA - Selective attention; E-ERQ - SS = Situation selection.*

7. **Moderation analyses, controlling for age in the case of female sample**
   7.1. **Moderation analysis using avoidant PD as the outcome**

**Table S11.** Moderation results for avoidant personality disorder (N = 125).

| Factor | b | SE  (HC3) | t | *p* | 95% CI | |
| --- | --- | --- | --- | --- | --- | --- |
|  |  |  |  |  | LL | UL |
| Hedonic goals | .052 | 0.498 | 0.104 | .916 | -0.935 | 1.040 |
| CTQ | .010 | 0.025 | 0.413 | .680 | -0.039 | 0.060 |
| Hedonic goals*CTQ | -.001 | 0.029 | -0.064 | .948 | -0.061 | 0.057 |
| Age | -.059 | 0.051 | -1.154 | .250 | -0.160 | 0.042 |
| Instrumental goals | 1.227* | 0.423 | 2.900 | .004 | 0.389 | 2.065 |
| CTQ | .010 | 0.025 | 0.416 | .677 | -0.039 | 0.060 |
| Instrumental goals*CTQ | -.009 | 0.026 | -0.365 | .715 | -0.061 | 0.042 |
| Age | -.047 | 0.047 | -0.994 | .322 | -0.142 | 0.047 |
| Reappraisal | -1.000* | 0.324 | -3.078 | .002 | -1.643 | -0.356 |
| CTQ | -.007 | 0.023 | -0.321 | .748 | -0.053 | 0.038 |
| Reappraisal*CTQ | .005 | 0.019 | 0.287 | .774 | -0.032 | 0.043 |
| Age | -.067 | 0.049 | 1.358 | .177 | -0.165 | 0.030 |
| Suppression | .914* | 0.295 | 3.095 | .002 | 0.329 | 1.500 |
| CTQ | .004 | 0.023 | 0.173 | .862 | -0.042 | 0.050 |
| Suppression*CTQ | -.001 | 0.016 | -0.093 | .925 | -0.034 | 0.031 |
| Age | -.064 | 0.053 | -1.198 | .232 | -0.171 | 0.042 |
| Distraction | .229 | 0.316 | 0.724 | .470 | -0.397 | 0.855 |
| CTQ | .010 | 0.025 | 0.408 | .683 | -0.039 | 0.059 |
| Distraction*CTQ | .0009 | 0.021 | 0.044 | .964 | -0.040 | 0.042 |
| Age | -.058 | 0.049 | -1.173 | .243 | -0.155 | 0.039 |
| Selective attention | .506 | 0.292 | 1.732 | .085 | -0.072 | 1.085 |
| CTQ | .014 | 0.024 | 0.568 | .570 | -0.034 | 0.062 |
| Selective attention*CTQ | -.012 | 0.015 | -0.768 | .443 | -0.043 | 0.018 |
| Age | -.054 | 0.048 | -1.123 | .263 | -0.150 | 0.041 |
| Situation selection | -.447 | 0.281 | -1.587 | .115 | -1.005 | 0.110 |
| CTQ | .003 | 0.025 | 0.141 | .887 | -0.046 | 0.053 |
| Situation selection*CTQ | -.018 | 0.018 | -0.963 | .337 | -0.055 | 0.019 |
| Age | -.064 | 0.049 | -1.295 | .197 | -0.162 | 0.034 |

**p ≤.05; CTQ = Childhood maltreatment.*

7.2. **Moderation analysis using dependent PD as the outcome**

**Table S12.** Moderation results for dependent personality disorder (N = 125).

| Factor | b | SE  (HC3) | t | *p* | 95% CI | |
| --- | --- | --- | --- | --- | --- | --- |
|  |  |  |  |  | LL | UL |
| Hedonic goals | -.045 | 0.611 | -0.075 | .940 | -1.257 | 1.165 |
| CTQ | .003 | 0.026 | 0.120 | .904 | -0.048 | 0.055 |
| Hedonic goals*CTQ | -.016 | 0.031 | -0.516 | .606 | -0.078 | 0.046 |
| Age | .052 | 0.058 | 0.902 | .368 | -0.062 | 0.167 |
| Instrumental goals | 1.629** | 0.465 | 3.500 | .0007 | 0.707 | 2.551 |
| CTQ | .004 | 0.024 | 0.179 | .857 | -0.044 | 0.053 |
| Instrumental goals*CTQ | -.024 | 0.025 | -0.944 | .346 | -0.074 | 0.026 |
| Age | .066 | 0.051 | 1.275 | .204 | -0.036 | 0.169 |
| Reappraisal | -.990* | 0.351 | -2.817 | .005 | -1.686 | -0.294 |
| CTQ | -.014 | 0.026 | -0.540 | .589 | -0.067 | 0.038 |
| Reappraisal*CTQ | .004 | 0.021 | 0.207 | .836 | -0.037 | 0.046 |
| Age | .042 | 0.056 | 0.757 | .450 | -0.068 | 0.154 |
| Suppression | .263 | 0.371 | 0.708 | .480 | -0.472 | 0.998 |
| CTQ | .001 | 0.026 | 0.046 | .962 | -0.050 | 0.052 |
| Suppression*CTQ | -.025 | 0.020 | -1.215 | .226 | -0.066 | 0.015 |
| Age | .055 | 0.060 | 0.915 | .361 | -0.064 | 0.174 |
| Distraction | .324 | 0.362 | 0.895 | .372 | -0.392 | 1.040 |
| CTQ | .003 | 0.025 | 0.124 | .901 | -0.048 | 0.054 |
| Distraction*CTQ | -.001 | 0.022 | -0.072 | .942 | -0.045 | 0.042 |
| Age | .052 | 0.056 | 0.931 | .353 | -0.059 | 0.165 |
| Selective attention | .587 | 0.344 | 1.703 | .091 | -0.095 | 1.269 |
| CTQ | .007 | 0.026 | 0.293 | .769 | -0.043 | 0.059 |
| Selective attention*CTQ | -.010 | 0.018 | -0.597 | .551 | -0.046 | 0.024 |
| Age | .056 | 0.055 | 1.010 | .314 | -0.053 | 0.165 |
| Situation selection | -.267 | 0.342 | -0.782 | .435 | -0.944 | 0.409 |
| CTQ | -.003 | 0.025 | -0.129 | .897 | -0.054 | 0.047 |
| Situation selection*CTQ | -.030 | 0.018 | -1.646 | .102 | -0.068 | 0.006 |
| Age | .053 | 0.056 | 0.945 | .346 | -0.058 | 0.164 |

**p ≤.05; **p ≤ .001; CTQ = Childhood maltreatment.*

7.3. **Moderation analysis using obsessive-compulsive PD as the outcome**

**Table S13.** Moderation results for obsessive-compulsive personality disorder (N = 125).

| Factor | b | SE  (HC3) | t | *p* | 95% CI | |
| --- | --- | --- | --- | --- | --- | --- |
|  |  |  |  |  | LL | UL |
| Hedonic goals | .567 | 0.426 | 1.332 | .185 | -0.275 | 1.411 |
| CTQ | -.005 | 0.017 | -0.311 | .756 | -0.040 | 0.029 |
| Hedonic goals*CTQ | -.066* | 0.026 | -2.503 | .013 | -0.118 | -0.013 |
| Age | -.026 | 0.034 | -0.764 | .446 | -0.094 | 0.041 |
| Instrumental goals | -.268 | 0.414 | -0.647 | .518 | -1.089 | 0.552 |
| CTQ | -.005 | 0.017 | -0.319 | .749 | -0.041 | 0.029 |
| Instrumental goals*CTQ | -.005 | 0.020 | -0.250 | .802 | -0.044 | 0.034 |
| Age | -.030 | 0.035 | -0.865 | .388 | -0.099 | 0.039 |
| Reappraisal | .321 | 0.269 | 1.191 | .235 | -0.212 | 0.854 |
| CTQ | -.0008 | 0.019 | -0.041 | .966 | -0.039 | 0.038 |
| Reappraisal*CTQ | -.017 | 0.022 | -0.797 | .426 | -0.061 | 0.026 |
| Age | -.023 | 0.035 | -0.651 | .516 | -0.093 | 0.047 |
| Suppression | .233 | 0.237 | 0.983 | .327 | -0.236 | 0.702 |
| CTQ | -.008 | 0.018 | -0.435 | .663 | -0.044 | 0.028 |
| Suppression*CTQ | -.008 | 0.013 | -0.661 | .509 | -0.034 | 0.017 |
| Age | -.027 | 0.034 | -0.795 | .427 | -0.096 | 0.041 |
| Distraction | .057 | 0.250 | 0.228 | .819 | -0.438 | 0.552 |
| CTQ | -.005 | 0.018 | -0.269 | .788 | -0.042 | 0.032 |
| Distraction*CTQ | -.017 | 0.017 | -0.999 | .319 | -0.051 | 0.016 |
| Age | -.024 | 0.035 | -0.678 | .498 | -0.094 | 0.046 |
| Selective attention | .238 | 0.250 | 0.951 | .343 | -0.257 | 0.734 |
| CTQ | -.005 | 0.018 | -0.271 | .786 | -0.041 | 0.031 |
| Selective attention*CTQ | -.011 | 0.016 | -0.693 | .489 | -0.045 | 0.021 |
| Age | -.025 | 0.035 | -0.735 | .463 | -0.095 | 0.043 |
| Situation selection | .459* | 0.218 | 2.099 | .037 | 0.026 | 0.892 |
| CTQ | -.001 | 0.016 | -0.078 | .937 | -0.034 | 0.032 |
| Situation selection*CTQ | .003 | 0.014 | 0.253 | .800 | -0.025 | 0.033 |
| Age | -.018 | 0.034 | -0.528 | .597 | -0.086 | 0.050 |

**p ≤.05; CTQ = Childhood maltreatment.*
